# A Multifunctional Tactile Sensory System for Robotic Intelligent Identification and Manipulation Perception

**DOI:** 10.1002/advs.202402705

**Published:** 2024-09-09

**Authors:** Yue Jiang, Lin Fan, Xilong Sun, Zehe Luo, Herong Wang, Rucong Lai, Jie Wang, Qiyang Gan, Ning Li, Jindong Tian

**Affiliations:** ^1^ Key Laboratory of Optoelectronic Devices and Systems of Ministry of Education and Guangdong Province College of Physics and Optoelectronic Engineering Shenzhen University Shenzhen 518060 China; ^2^ College of Computer Science and Software Engineering Shenzhen University Shenzhen 518060 China; ^3^ Guangdong Laboratory of Artificial Intelligence and Digital Economy (Shenzhen) Shenzhen University Shenzhen 518132 China; ^4^ Institute of Applied Physics and Materials Engineering University of Macau Macao 999078 China

**Keywords:** interaction state perception, lotus nanofiber, multi‐feature recognition, multimodal tactile, MXene

## Abstract

Humans recognize and manipulate objects relying on the multidimensional force features captured by the tactile sense of skin during the manipulation. Since the current sensors integrated in robots cannot support the robots to sense the multiple interaction states between manipulator and objects, achieving human‐like perception and analytical capabilities remains a major challenge for service robots. Prompted by the tactile perception involved in robots performing complex tasks, a multimodal tactile sensory system is presented to provide in situ simultaneous sensing for robots when approaching, touching, and manipulating objects. The system comprises a capacitive sensor owning the high sensitivity of 1.11E‐2 pF mm^−1^, a triboelectricity nanogenerator with the fast response speed of 30 ms, and a pressure sensor array capable of 3D force detection. By Combining transfer learning models, which fuses multimodal tactile information to achieve high‐precision (up to 95%) recognition of the multi‐featured targets such as random hardness and texture information under random sampling conditions, including random grasp force and velocity. This sensory system is expected to enhance the intelligent recognition and behavior‐planning capabilities of autonomous robots when performing complex tasks in undefined surrounding environments.

## Graphical abstract

In this paper, an artificial intelligence‐motivated, multimodal tactile sensory system is presented, in which the multiple sensors are entirely prepared from the MXene/lotus nanofiber‐contained composite films, to provide in situ simultaneous sensing for robots when approaching, touching, and manipulating objects. By Combining transfer learning models, which fuses multimodal tactile information to achieve high‐precision recognition of the multi‐featured targets under random sampling conditions.

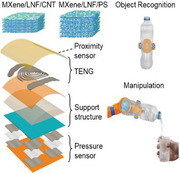

## 1 Introduction

Service or collaborative robots are often required to perform complex tasks involving unstructured targets in undefined environments,^[^ ^1^ ^]^ as yet such tasks are challenging for robots. For instance, it is generally simple for a robot to perform a grasping action, but how to accurately recognize a target object in an unknown environment, and then perform a firm and stable grasp and execute a manipulation without destroying the object is complicated. For humans, utilizing multiple tactile receptors in the skin (e.g., slow‐adaptive and fast‐adaptive receptors) not only allows dexterous manipulation, but also incorporates a priori knowledge to distinguish multiple features of objects such as contours, materials, and textures of objects.^[^ ^2^ ^]^ Robots rely on the tactile perception in manipulator‐object interactions to make intelligent decisions and perform dexterous manipulation. So, a human‐like tactile sensory system is essential for robots.^[^ ^3^ ^]^

Robots utilize the electronic skin (e‐skin) for tactile sensing to achieve the real‐time perception of robot‐object interaction states.^[^ ^4^ ^]^ Bao et al. proposed a tactile sensor based on a pyramidal microstructure with a bionic leaf‐like helical arrangement that can provide static pressure feedback when grasping an object.^[^ ^5^ ^]^ Arias et al. enabled the detection of complex dynamic pressures by developing a single‐modal self‐powered mechanoreceptor used as a soft robot gripper.^[^ ^6^ ^]^However, the loading of such single‐modal e‐skins only offer the independent pressure feedbacks of static or dynamic stimulus for robots, which is not favorable for the robots to establish the timing and logic of sensory.^[^ ^7^ ^]^

The machine learning‐motivated e‐skins can recognize a wide range of targets and avoids the inherent limitations of vision technology, such as difficulty in perceiving transparent objects and interference from the ambient light.^[^ ^8^ ^]^ For instance, Jin et al. demonstrated a smart soft‐robotic gripper system comprising patterned‐electrode tactile sensors and gear‐structured length sensors, the system accomplished the recognition of various objects by machine learning.^[^ ^2^ ^]^ Sundaram et al. demonstrated a scalable tactile glove based on a tactile sensor array.^[^ ^9^ ^]^ By means of the post‐processing of tactile information with deep convolutional neural networks, this glove was endowed with the intelligent recognition of objects and the estimation of object weight. However, the coupling of multiple features of the objects (e.g., softness, hardness, texture, material, temperature, etc.) severely degrades the object recognition accuracy of robots based on the single‐modal tactile features.^[^ ^10^ ^]^

When a human grasps an object, the weight, size, temperature, and texture of the object, and even the manipulation process are all perceived simultaneously, owning to the synergistic processing of multiple tactile sensory information by the brain.^[^ ^11^ ^]^ Therefore, developing a multimodal tactile sensory system capable of capturing multidimensional tactile information from a single interaction action can effectively enhance the intelligent recognition and dexterous manipulation ability of robots.^[^ ^12^ ^]^ Herein, we present a somatosensory system‐inspired, artificial intelligence‐motivated, multimodal tactile sensory system capable of material recognition and interaction state perception. The developed tactile sensory system has a hierarchical structure comprising a proximity sensing and triboelectricity nanogenerator (TENG) coplanar layer, and a pressure sensing array layer, all of which are formed by the patterned functional films containing MXene and lotus nanofibers (LNF) extracted from the natural lotus silk. Furthermore, we demonstrate that the fusion of multimodal tactile sensing information can effectively improve the recognition accuracy of targets with different hardness, material, and texture characteristics, and clearly reveal the details of the manipulator‐object interactions even during highly randomized grasping or manipulation when the robot is performing complex tasks. The proposed multimodal tactile sensory system demonstrates considerable potential in promoting the intelligent perception and behavior planning capabilities of service robots in diverse scenarios.

## 2 Results and Discussion

### 2.1 Concept of the Multifunctional Tactile Sensory System

**Figure 1:**
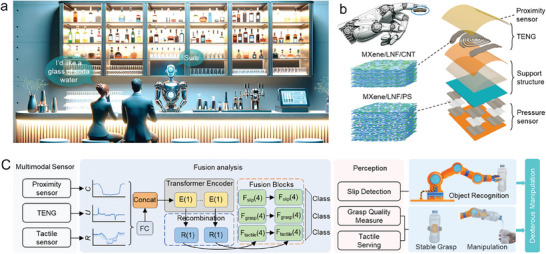
Concept and design of a multimodal tactile sensory system for intelligent tasks. a) In the common application scenario where a service robot serves customers, the robot needs to accurately recognize the drinks required by the customers and complete a series of dexterous operations including picking up glasses and pouring drinks into the glasses. The multimodal tactile sensory system can provide the robot with physical information of objects and manipulator‐object interaction details to assist the robot to perform these complex tasks. b) Structural illustration of the multimodal tactile sensor based on MXene/LNF composites. c) The multimodal sensing signals are fused and analyzed by artificial intelligence algorithms to provide support information for intelligent recognition and dexterous manipulation of the robot.

In the common application scenario where a service robot serves customers, the robot needs to accurately recognize the drinks required by the customers and complete a series of dexterous operations including picking up glasses and pouring drinks into the glasses (**Figure** **1**a). The robot needs to achieve object recognition, independent planning, and dexterous manipulation, supported by the tactile sensory system for providing personalized services.^[^ ^13^ ^]^ The multimodal sensor consists of a coplanar distributed TENG and capacitive sensor with helical electrodes, and an array of pressure sensors utilized to detect the pressure distribution patterns induced by 3D force during contact with targets (Figure 1b). The flexible conductive electrodes (MXene/LNF/CNT), and the flexible pressure‐sensitive layers (MXene/LNF/PS) with micropores and microstructures are fabricated from compositing the LNF with MXene, carbon nanotube (CNT), and polystyrene microsphere (PS), respectively. A fusion model for multimodal tactile information is proposed to support robots in realizing recognition of multiple targets (even transparent objects) and multi‐feature perception of manipulation‐object interaction (Figure 1c).

### 2.2 Characterizations and Properties of Composite Films

**Figure 2:**
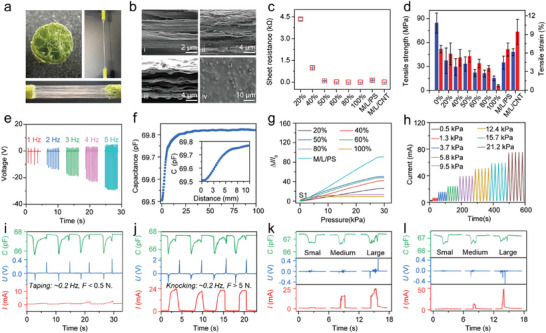
Characterizations of the functional films and sensing properties of multimodal sensors. a) Macroscopic morphology of the lotus filament. b) SEM characterizations of the cross‐sectional view of the films: i) MXene/LNF, ii) MXene/LNF/CNT, iii) MXene/LNF/PS; and iv) the surface of MXene/LNF/PS film. c) Sheet resistance of the films. d) Typical tensile strength and tensile strains of the films. e) The output voltage of the TENG responding to the applied pressure at different frequencies. f) The changes in capacitance of the capacitive sensor with the distance between the sensor and the target. g) The responses in current of the various function films to the increasing pressure. h) the four cycles of response in current of the MXene/LNF/PS film‐based pressure sensor during the step loading and releasing of pressure. i,j) The sensing signals of the multi‐parameter sensor responding to the tapping and knocking actions, respectively. k,l) The sensing signals of the multimodal sensor during performing a sliding action with different pressures on the substrates with (k) abundant granular bumps and (l) regularly striped bumps, respectively.

As a new 2D nanomaterial, MXene exhibits high specific surface area, high conductivity, and excellent flexibility, which is considered to have great application prospects in a variety of flexible sensors.^[^ ^14^ ^]^ Pure MXene films exhibit drawbacks, including poor mechanical strength, easy self‐stacking, and insufficient oxidation resistance.^[^ ^15^ ^]^ Lotus silk, derived from the stem of lotus flowers, stands as a natural fiber (**Figure** **2**a). Employing the Tempo method, we have successfully prepared lotus silk nanofibers for the first time.^[^ ^16^ ^]^ TEM characterizations (Figure S1a and S1b) show that Ti_3_C_2_ nanosheets prepared from Ti_3_AlC_2_ raw material and that the uniformly dispersed LNF have the diameter of ≈ 10−15 nm and a length of ≈ 500 nm. This uniform distribution of both MXene and LNF in the water indicates favorable water dispersion characteristics (Figure S1c, Supporting Information).^[^ ^17^ ^]^

In Figure S6 (Supporting Information) and Experimental section, MXene is mixed with LNF in varying ratios using ultrasonic and magnetic stirring techniques. Subsequently, vacuum filtration is performed to produce the MXene/LNF composite film. The films’ cross‐sectional SEM characterizations reveal the ordered layer structure of the composite films with different ratios (Figure S2, Supporting Information). Through elemental mapping (Figure S3, Supporting Information) and XPS spectra (Figure S4, Supporting Information), the uniform distribution of LNF within the composite films is obvious. FTIR analysis (Figure S5, Supporting Information) indicates the oxygen‐containing functional groups in the composite films are significantly increased, meanwhile, hydrogen bonds are formed.^[^ ^18^ ^]^ The tensile strength and strain characteristics of the films are illustrated in Figure 2d and S8. Obviously, the MXene/LNF composite films exhibit excellent mechanical strength and toughness, even though the mass ratio of MXene reaches more than 50%. This is mainly attributed to the self‐assembly behavior between the abundant functional groups (e.g., ─O─, and ─OH) on the MXene nanosheets and the reactive hydroxyl groups (─OH) on the LNF.^[^ ^19^ ^]^ Furthermore, the LNF have a fiber bundle structural feature similar to reptile tentacles, which can effectively anchor the nanosheets to form a stable laminar structure without significantly weakening the contact properties between the MXene nanosheets.^[^ ^20^ ^]^ Figure 2c shows that the conductivity of the composite film with a mass ratio of 50% MXene is similar to that of the pure MXene film. As shown in Figure S9 (Supporting Information), the MXene/LNF composite film is completely degraded after 14 days in 1wt.% H_2_O_2_ solution. In a strong oxidizing environment, hydroxyl and other functional groups are oxidized, which reduces the degree of polymerization of LNF, and MXene is also easily decomposed.^[^ ^21^ ^]^

In the pursuit of enhancing the pressure sensitivity of pressure sensors, increasing the micropores within the pressure‐sensitive film is an effective approach.^[^ ^22^ ^]^ PS microspheres (Figure S1d) were introduced into the pumped precursor solution to produce MXene/LNF/PS composite films (weight ratio, 20:1). The SEM morphology image (Figure 2b. iii) highlights that PS microspheres endow the composite film with ample interstitial space while creating abundant bulge structures on the film surface. The XRD spectra (Figure S7, Supporting Information) shows that the characteristic peak (002) of MXene film gradually shifts to the left after mixing with LNF and PS, indicating that the introduction of 1D LNF and PS microspheres increases the interlayer spacing of the nanosheets. MXene/LNF/CNT composite film exhibits a more densely packed stacking structure, and CNT fibers are extensively distributed throughout the film, spanning numerous MXene nanosheets (Shown in Figure 2b ii).

### 2.3 Performance of the Fabricated Multifunctional Sensor

The electrode structures of TENG are composed of the laser‐pattered MXene/LNF/CNT composite film, which is transferred onto an ultrathin PDMS substrate. The electrode surface is coated with nylon material in the molten state (Model 12, melting temperature 175 °C), which acts both as a triboelectric layer for the TENG and as a protective layer for the capacitive sensor.^[^ ^23^ ^]^ The TENG operates on a single electrode sampling mode. Upon contact and separation from a target, surface charge transfer occurs due to the disparity in electron affinity (Figure S10, Supporting Information). TENG exhibits ultra‐fast response speed (The response and recovery time of TENG is 38 and 31 ms, respectively. Figure S11a, Supporting Information) and excellent consistency (Figure S11b). Consequently, the TENG accurately and rapidly characterizes the contact behavior between the TENG and the target. Figure S12 (Supporting Information) showcases *V*−*t* curves at the applied pressures below 5 kPa, revealing an increase in output voltage with the rising applied pressure. Each curve exhibits two opposing peaks during contact and release. In Figure 2e, a series of stable and continuous *V*−*t* curves for the different frequencies of pressure indicate the amplified voltage when the TENG operates at a higher frequency of 5 Hz. These characteristics highlight the significant influence of the applied pressure on output voltages.^[^ ^24^ ^]^ The voltage amplitude of the TENG is slightly affected by temperature (Figure S14a, Supporting Information), which is attributed to the fact that the charge movement of the object is more intense at higher temperatures, which enhances the charge interaction between the TENG with the objects. The voltage amplitude of the TENG is inversely proportional to humidity (Figure S15a, Supporting Information), which is attributed to the fact that the spatial depletion of surface charges on the object caused by high‐humidity air interferes with the charge interaction between the TENG interface and the objects. The output voltage of the TENG decreases with increasing curvature of the film, due to the fact that the larger curvature makes the smaller contact area between the sensor and the object (Figure S16a, Supporting Information).

The capacitive sensor has the twin helical coils that generate an alternating electromagnetic field perpendicular to the electrodes under high‐frequency excitation. The capacitance response of the sensor is mainly attributed to the perturbation of the alternating electromagnetic field caused by the approaching objects. Thus, the capacitive sensor is capable of detecting various conductors, beverages, and human hands with acceptable consistency (Figure S17b, Supporting Information). As shown in Figure 2f, it has a detection distance exceeding 5 cm and the higher accuracy in the range of 0−5 mm (Distance sensitivity of 1.11E‐2 pF mm^−1^, Figure S17a, Supporting Information). Also, the response of the sensor is positively correlated with the conductivities of objects (Figure S18, Supporting Information). The responses of the capacitive sensor are insensitive to the changes in temperature (Figure S14b, Supporting Information). But it is proportional to humidity (Figure S15b, Supporting Information), because the high‐humidity air has a higher dielectric constant, and the change in dielectric properties of the air between the sensor and the objects is more pronounced during proximity. The response of the capacitive sensor decreases with increasing curvature of the shim (Figure S16b, Supporting Information), due to the bending of the film increasing the EMF dissipation of the capacitor.

The pressure sensor is constructed by utilizing the MXene/LNF/CNT composite films as the upper and lower electrodes, while encapsulating the MXene/LNF/PS composite films as the pressure‐sensitive layer. In Figure S19 (Supporting Information), the *I*−*V* curves of the sensors show the obvious linearity, and the curve slopes exhibit a positive correlation with the applied pressure, indicating the establishment of an ohmic contact between the electrode layer and the pressure‐sensitive layer. Employing the fatigue machine, the dynamic pressing cycles ranging from 0–30 kPa is generated, with an indenter movement speed of 0.5 mm min^−1^. As depicted in Figure 2g, the pressure sensor demonstrated a more linear pressure‐sensitive response within the 30 kPa range. The sensitivity of the MXene/LNF/PS film‐based pressure sensor is 4.34 kPa^−1^ (Figure S20a, Supporting Information). The responses of the pressure sensors utilizing the MXene/LNF composite films with different ratios as the pressure‐sensitive layer reveal that the introduce of PS microsphere obviously enhances the pressure‐sensitive performance of the sensors. This enhancement primarily stems from the abundant gaps distributed within the film after the introduce of PS microsphere. Additionally, the formation of micro‐bump structures on the surface contributes to a notable structural interlocking effect within the pressure‐sensitive layer.^[^ ^25^ ^]^

A periodic step pressure cycling process is applied to the pressure sensor, showcasing the sensor's response cycle in Figure 2h. Comparing with the cyclic output curves of the pressure sensors using pure MXene pumped film as the sensitive layer (Figure S21, Supporting Information), the pressure sensor using MXene/LNF/PS film as the sensitive layer displays an improved long‐term stability (Figure S22, Supporting Information). This improvement stems from the establishment of binding sites between LNF nanofibers and MXene nanosheets, such as hydrogen bonds. Furthermore, this pressure sensor exhibits a fast response and recovery time of 85 ms and 83 ms, respectively (Figure S20b, Supporting Information). The consistency of the pressure sensor has been shown in Figure S20c (Supporting Information). The maximum responses of the pressure sensor in the current increase with temperature (Figure S14c, Supporting Information). Due to the protection of the encapsulation layer, the response of the pressure sensor remains stable under different environment humidities (Figure S15c, Supporting Information). The response of the pressure sensor is proportional to the curvature of the shim, owing to the greater intensity of pressure induced by the smaller contact area between the objects and the sensor attached to the shim with the larger curvature (Figure S16c, Supporting Information). Figure S23 (Supporting Information) demonstrates the response of the pressure sensor to the fine deformation induced by human pulse waves. Simultaneously, Figure S24 (Supporting Information) validates the high sensitivity of the pressure sensor in detecting human activity, such as finger bending, wrist bending, and elbow swing.

The comprehensive measurements were conducted for a multi‐parameter sensor consisting of a capacitive sensor, a TENG, and a pressure sensor, to evaluate the synergy of the three kinds of sensors when integrated into a monolithic device. In the measurements, two distinct actions: “touch” and “press”, applied by a machine, are employed, where the “touch” and “press” actions refer to the applied pressure of less than or equal to 3 N, and greater than or equal to 13 N, respectively. As depicted in Figure S25 (Supporting Information), the response signal generated by the piezoresistive component is weak during the “touch” action, due to the low pressure. However, the capacitive signals exhibit the prolonged characteristics during approach and departure, meanwhile, the triboelectric signals exhibit the parametric features of contact and separation. Even when rapid movements are employed, such as “tapping” and “knocking” actions are applied to the substrate when the finger wears the sensor, the intricacies of the action are distinguished well from the three output signals (Figure 2i,j). This multi‐parameter sensor plays a crucial role in distinguishing the different actions of human activity.

To effectively mimic the daily interaction scenarios, the fingertip wearing a hybrid sensor perform sliding actions with different pressures on a substrate with abundant granular bumps and the other substrate with regularly striped bumps, respectively. As the response, the multi‐parameter sensor generated three output signals with different features (Figure 2k,l). These measurements illustrate that the multi‐parameter sensor effectively provides proximity, contact, slip, and pressure perception during the interactions between the manipulator and the target.

### 2.4 Multi‐Feature Recognition Strategy of the Multifunctional Tactile Sensory System

**Figure 3:**
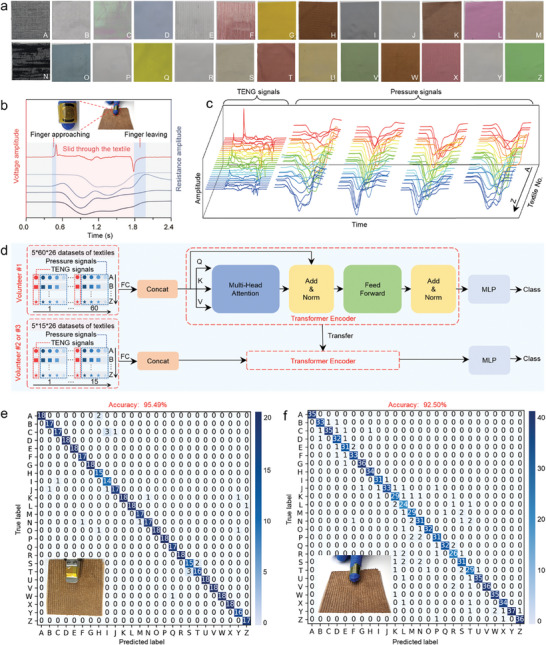
Multi‐feature recognition strategy of the multifunctional tactile sensory system. a) The digital photos of 26 different textiles. b) and c) The pressure and triboelectric signal characteristics of 26 textiles utilizing the same sliding action for sampling. d) The detailed framework of the transfer learning model. e) Confusion matrix of object recognition derived from transfer learning model using the database of triboelectricity‐pressure signals acquired by manipulator sampling. f) Confusion matrix of object recognition based on manually sampled triboelectricity‐pressure signal datasets.

The difference in the intrinsic ability to capture and lose electrons between the contact materials determines the polarity and magnitude of the triboelectric signals.^[^ ^26^ ^]^ Currently, TENG‐based recognition strategies usually require robots to repeatedly grasp objects to compile training and test datasets.^[^ ^27^ ^]^ However, variations in shape, texture, and hardness of objects lead to random and unpredictable contact characteristics with the sensor. For example, the features of triboelectric signals are quite different (Figure 3b,c), when the manipulator grasps the textiles with a soft texture and distinctive texture features (**Figure** **3**a; Table S1, Supporting Information). Objects are often placed in an overlapping manner in daily scenarios. For example, the feelings of stroking the same blanket placed on a table or on a soft bed are distinct. To mimic this scenario, the textiles are placed on three plates with different hardnesses (acrylic plastic, silicone, and sponge). The sensor is installed on the fingertip of the manipulator, that follows a fixed contact‐sliding‐detachment motion to stroke the textiles in three different directions. A dataset is constructed using the output signals of the TENG and four pressure sensors. 7800 datasets are collected for 26 textiles, 20% of which are used for testing.

In this study, A transfer learning model is implemented for the dual‐modal signal processing (Figure 3d and Experimental section). The recognition accuracy based on the pressure‐triboelectricity signals reaches up to 95.49% (Figure 3e), but the recognition accuracy based on the single triboelectric signals is 76.62% (Figure S26, Supporting Information). When the fingertip of Volunteer #1 loads the sensor and performs a stroking action similar to that of the manipulator, the significant fluctuations in the output signals of the sensor are observed owing to the randomness of the applied force and the motion speed during sampling by hand (Figure S27, Supporting Information). Obviously, the recognition accuracy based on the pressure‐triboelectricity dual‐modal signals (92.5%, Figure 3f) demonstrates the significant improvement compared with the recognition accuracy based on the single triboelectricity signals (52.23%, Figure S28, Supporting Information). These results highlight that combining multimodal tactile sensing information effectively enhances the recognition accuracy of targets with different hardness, materials, and textures. In addition, it has greater redundancy for sampling data fluctuations caused by the changes in the applied force and the operating speed during grasping or manipulation. Two dual‐modal sensors are loaded on the fingertips of two other volunteers (#2 and #3), respectively. Then, the volunteers stroke the 26 textiles with the action similar to the manipulator. The newly collected datasets from each of two volunteers (1560 data sets) is only 20% of the datasets from Volunteer #1 and is merged with the original datasets (7800 datasets for the 26 textiles). The transfer learning model is able to capture context information for global relationship modeling when processing the sequential data, rather than merely obtaining local features. Based on the newly collected datasets from each of Volunteer #2 and #3, the recognition accuracies of the different textiles are 85.8% and 84.2%, respectively (Figure S29, Supporting Information).

### 2.5 Interaction State Perception of Manipulators

Robotic manipulators extensively showcase the capability to handle soft or delicate objects. In this study, a multimodal sensor is mounted on the fingertip of the manipulator. From the top to the bottom, the multimodal sensor consists of the TENG and capacitive sensor co‐planarly arranged on a finger‐like semi‐cylindrical soft structure, and the array of four pressure sensors distributed under a rigid substrate for accurately detecting the uneven pressure distribution features induced by 3D force.

First, we have performed the experiments on the responses of a pressure sensor array to the regularly varying 3D force. As shown in Figure S30b (Supporting Information), ***F***_xoy_ is the projection of the 3D force ***F*** in XOY plane, *α* is the angle between ***F***_xoy_ and ***F***, *θ* is the angle between ***F***_xoy_ and X‐axis, and *f* is the magnitude of ***F***. So, the 3D force ***F*** can be expressed as {*f*, *α*, *θ*}. When *f* = 10 N, *θ* = 45° and *α* is from 30° to 150°, the difference in resistance responses of the four sensors are shown in Figure S31a (Supporting Information); When *f* = 10 N, *α* = 45° and *θ* is from 0° to 360°, the resistance responses of the four sensors are showed in Figure S31b (Supporting Information). It is proven that the responses of the pressure sensor array can accurately characterize a 3D force.

**Figure 4:**
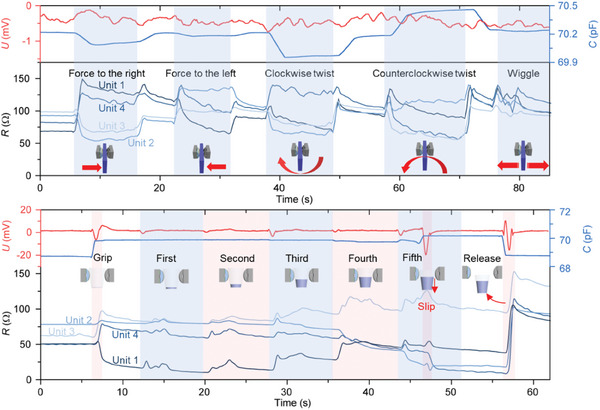
Multi‐feature perception using the multimodal sensor in the execution of a particular operation by robot. a) the multi‐feature perception when the robot grips a plastic plate applied with different kinds of pressures from the fingers. The pressures include the force on the left and the right, the twisting force in the clockwise and counterclockwise directions, and the force generated from wigging the plate. b) The multi‐feature perception when the robot grips a plastic cup along with the repeated operation of adding water into the plastic cup by another cup (60 mL) filled with water.

As depicted in **Figure** **4**a, when the manipulator grips the plastic plate, we apply pressure with fingers in different directions: twisting it clockwise or counterclockwise, or wiggling the other side of the plate. When the manipulator grips the plastic plate, the elastic support structure of the sensor is compressed, resulting in the deformation of the four pressure sensors, corresponding to the initial state of the resistance (Figure S32a, Supporting Information). When a horizontal force is applied, the plastic plate undergoes deflection in the direction of the force (Figure S32b, Supporting Information). The elastic support structure of the sensor deforms, i.e., the near‐end is compressed and the far‐end is released, and consequently, the force applied on the four pressure sensors changes, resulting in the changes in the resistance. When a twisting force is applied, the plastic plate is deflected along the center of the twisting force (Figure S32c, Supporting Information). The contact position between the plastic plate and the sensor is shifted. The force position and the shape of the elastic support structure change, which affects the force applied on the pressure sensors. The output signals of the four pressure sensors accurately reflect the changes in force applied to the plastic plate. The capacitive sensors also have the rapid response attributed to the deformation of the elastic support structures induced by the applied force. The triboelectric signals do not exhibit the noticeable voltage spikes, indicating that the plastic plate does not separate from the sensors during the force application.

To further illustrate the capability of the multimodal sensor in handling soft and slippery objects, the experiment involving the manipulation of an empty plastic cup with the same control strategy was conducted. As depicted in Figure 4b, A small cup (60 mL) filled with water is manipulated to repeatedly add water into a paper cup. The changes in the force of the four sensors during the process of pouring water into the cup are shown in Figure S33 (Supporting Information). The static friction between the wall of the cup and the sensor increases with increasing water volume. At the same time, the soft cup undergoes compressive deformation due to gravitational drop along with the change in contact position. As a result, the force distributed on the four sensors changes. As water is added, the cup is shocked by the water falling from the height, which induces the sharp fluctuation of the pressure sensor signals compared with the relatively stable state before water addition. Simultaneously, the TENG responds to the weak vibrations of the cup caused by the addition of water. Moreover, the capacitive sensor exhibits an obvious response when the electromagnetic field is disturbed by the addition of water, particularly when the water volume approaches or overs the clamping position. With the gradually increasing in the amount of water, the progressive changes occur to the signals of the pressure sensors. Eventually, when the balance between the gravity of the cup with water and the static friction is broken, the cup slides down. The TENG demonstrates the real‐time response to the sliding process.

The above experiments demonstrate the excellent capabilities of the developed multimodal tactile sensor in sensing the proximity, contact, gripping pressure, and shear force in real‐time. The multi‐parameter‐involved sensing information provides an effective support for stable and dexterous manipulation of the robots, which is extremely useful for robots to perform precision operation in unstructured environments.

### 2.6 Intelligent Identification and Manipulation Perception in Robot

**Figure 5:**
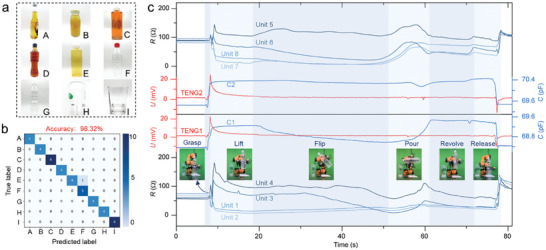
Intelligent identification and manipulation perception using the multifunctional tactile sensory system. a) Digital photos of 9 objects (A: beer, B: juice, C: green tea, D: red tea, E: white tea, F: pure water, G: plastic bottle, H: plastic cup, I: glass cup). b) Confusion matrix of object recognition based on capacitance‐triboelectricity‐pressure signal datasets. c) Multi‐feature perception using the multimodal sensor when pouring water into a plastic cup.

Robots equipped with multi‐functional haptic perception systems have the ability to intelligently recognize and manipulate perceptions, and are more adept at performing realistic tasks. As a conceptual demonstration, the robot is required to recognize the water bottle and plastic cup from 9 common household items (**Figure** **5**a) and perform a water pouring task. After initial sampling and training, the recognition accuracy of the 9 target objects reaches up to 98.32% (Figure 5b). During the subsequent pouring process, the multimodal tactile sensor demonstrates the responses to the changes in proximity, contact, pressure, and shear force, guiding the robot action planning during the operation (Figure S34, Supporting Information). When the manipulator performs the water‐pouring action, the force on the sensor array in different postures is shown in Figure S35 (Supporting Information). As the bottle is flipped, the force on the sensor array changes following the offset of the gravity center of the bottle. The pressure sensing arrays of two multimodal sensors loaded on each of the robot's fingertips, respectively (Figure 5c; Movie S1, Supporting Information), accurately reflect the multidimensional forces applied to the fingertips. The capacitance fluctuations of the two capacitance sensors caused by the water flow approaching or flowing through the gripping position are accurately captured. All of this information can be analyzed to predict the position changes of contents in the objects. In summary, the developed multifunctional tactile sensory system assists robots in the manipulation of unstructured objects.

## 3 Conclusion

In this study, LNF extracted from natural lotus silk is introduced into MXene films to enhance the flexibility of composite films. Diverse functional films (MXene/LNF/PS and MXene/LNF/CNT) are further prepared by incorporating different materials. The proposed multimodal tactile sensor perceives proximity, contact, gripping pressure, and shear force in real‐time, which enhances the fine manipulation capability of robots. The transfer learning model based on multimodal tactile information realizes the high‐precision recognition of multi‐feature coupled targets. Furthermore, the transfer capability of the constructed model significantly reduces the scale of data sampling. The proposed multifunctional tactile sensory system is expected to be used for novel service robots and collaborative robots.

## 4 Experimental Section

### Materials

Lotus stems were collected in a natural environment. Ti_3_AlC_2_ powder (200 mesh) was purchased from 11 Technology Co., Ltd. (China). Lithium fluoride (LiF, AR) was purchased from Shanghai Macklin Biochemical Technology Co., Ltd. (China). Hydrochloric acid (HCl, 37%) was purchased from Chengdu Huacheng Chemical Co., Ltd. (China). Laboratory grade 2,2,6,6‐tetramethylepiperidin‐1‐oxyl (TEMPO), sodium bromide (NaBr), 13% sodium hypochlorite (NaClO) solution, sodium hydroxide (NaOH), and ethanol (C_2_H_5_OH) were purchased from Shanghai Aladdin Biochemical Technology Co., LTD. PS microsphere was supplied by Dongguang Kemai New Material Co., Ltd. CNTs solution (diameter 5–15 nm) was obtained from Jiacai Technology Co., Ltd. All chemicals were used without further processing.

### Synthesis of Ti_3_C_2_ MXene

Ti_3_C_2_ MXene flakes were synthesized by etching the aluminum layers of Ti_3_AlC_2_ in a fluoride‐containing acidic solution. LiF (2 g) was added into HCl (40 mL, 9 m) and stirred for 10 min. Ti_3_AlC_2_ MAX phase (2 g) was gradually added into the solution with an ice bath, and the mixture was stirred for 24 h. Subsequently, the reacted solution was centrifuged at 3500 rpm for 10 min, the upper clear solution was poured off and the deionized water (30 mL) was added. This process was followed by sonification for 5 min. These processes were repeated until the pH of the upper solution was neutral. After centrifuging, ethanol (40 mL) was added to the solution, followed by sonification for 30 min to collect the product. The residual ethanol in the product was washed off by deionized water and centrifugation (10000 rpm, 10 min). The product was mixed with deionized water (20 mL) again. After shaking and sonification, the mixture was centrifugated (10000 rpm, 10 min) and the upper clear solution obtained was the Ti_3_C_2_ dispersion. The sonification and centrifugation processes were repeated to collect more Ti_3_C_2_ dispersion. The concentration of Ti_3_C_2_ in the dispersion was measured by weighing the film prepared via the vacuum filtration method.

### Synthesis of LNF

Pre‐treatment of raw materials: first, the lotus silks were shortened to 3−5 mm, then alkali treated it with 20% concentration of sodium hydroxide for 30 min. The treated lotus fibers were washed with deionized water to the neutral pH value, further air‐dried, and weighed. TEMPO‐mediated oxidation: the extracted fibers were configured into a slurry concentration of 0.8% (w/v) with deionized water in a 2 L reactor container, then the appropriate amount of NaBr, TEMPO was added and further homogenized with ultrasonic treatment for 10 min. An amount of NaClO solution was added during the reaction using a peristaltic pump at room temperature by stirring at 500 rpm. The PH of the reaction was maintained at 10 by continuous addition of 0.5 m NaOH using a PH stat until no NaOH consumption was observed. The reaction time for 1 h and then the reaction was terminated by the addition of ethanol solution. The oxidized lotus fibers were purified by thoroughly washing with deionized water. After centrifugation and purification to neutrality, the washed product was then suspended in water and further dispersed into nanofibers suspension (0.6%), which was homogenized by a high‐pressure homogenizer at 100 MPa for 6 times to obtain a LNF suspension.

### Preparation of MXene/LNF, MXene/LNF/PS and MXene/LNF/CNT Composite Films

The composite films were prepared via a simple vacuum‐assisted filtration method. The MXene, LNF, PS microsphere, and CNT aqueous dispersion at a concentration of 4 mg ml^−1^, respectively. The mixture of MXene, LNF, PS, and CNT was sonicated 20 min and stirred for 1 h to form a uniform hybrid suspension. Then, the mixture was filtered through a PES membrane to produce composite films that were dried in a vacuum oven for 4 h at 50 °C. The weight ratios of MXene to total quantity (MXene/LNF) were 0%, 20%, 40%, 50%, 60%, 80% and 100%. In addition, MXene/LNF/PS, MXene/LNF/CNT, pristine MXene, and LNF films were also produced above the same conditions. Then, the free‐standing films were peeled from the filter membrane to obtain hybrid films and tailored to different patterns by laser for further device preparation.

### Characterization and Testing

The structures, morphologies, and elemental mappings of MXene nanosheets, LNF, and composite films were characterized by Transmission electron microscopy (TEM) (JEM‐1200EX, JEOL, Japan) and Scanning electron microscopy (SEM) (SU8020, Hitachi, Japan). X‐ray diffraction (XRD) patterns of the films were recorded from a Bruker D8 AVANCE instrument, the Fourier transform infrared spectroscopy (FTIR, Nicolet 6700) instrument was applied to detect the physical interaction of the hybrid films in the range of 400−4000 cm^−1^. XPS spectra measurements were performed using an X‐ray photoelectron spectrometer (ESCALAB 250Xi, Thermo Scientific). Mechanical properties of the films were characterized by using a testing machine (ZQ‐950B, Zhiqu Precision Instrument Co., Ltd. Dongguan, China). Each sample was cut into a strip of 10 mm × 25 mm, and the loading rate was 0.5 mm min^−1^. The electrical properties of the films were characterized by using a four‐point probe instrument, and the data recorded at 10 positions were averaged to characterize the electrical properties. The sensing performance of the hybrid films pressure sensor was recorded on the compression testing machine and a source meter (Keithley 2450), while the input constant voltage was 0.5 V during the tests. The four pressure sensors resistance curves were recorded by a multi‐channel multimeter system (Keithley DAQ6510). The output voltage of the TENG was measured using a digital oscilloscope (Tektonix, MSO56). The output capacitive signal of the proximity sensor was acquired using an LCR meter (HIOKI, IM 3536).

As a Pico ammeter, the source meter (Keithley 2450) capable of providing a low‐noise voltage can accurately measures the weak changes in the current of the pressure sensors responding to small pressure. So, the current of pressure sensors is suitable for accurately characterizing the sensitivity of single‐modal pressure sensors in Sub‐section 2.3. When sampling the responses of the pressure sensor array to the applied pressure, Keithley 6510 as an arrayed data acquisition unit is able to characterize the response in the resistance of the sensor array to pressure. Therefore, the resistance is chosen as an output indicator after the Sub‐section 2.3.

The experimental setup used to evaluate the sensing performance of the pressure sensor at different temperatures is shown in Figure S13a (Supporting Information). A silicone rubber heating plate used to control the temperature is placed on the back of the pressure sensor. The experimental setup used to evaluate the sensing performance of the capacitive sensor and TENG sensor at different temperatures is shown in Figure S13d (Supporting Information). Because the eddy current generated by the electrode inside the heating plate interferes with the response of the capacitive sensor, a copper sheet is used as a heat conductor to change the temperature of the sensor. In order to reduce the noises, a grounded copper sheet is placed between the heating plate and the TENG as a shielding layer. The experimental setup used to evaluate the sensing performance of the sensors under different humidities is shown in Figure S13b (Supporting Information). High humidity air is obtained when dry air is moistened by passing through a number of bottles with water. The humidity of the air is adjusted by changing the flow rate of the air and the temperature of the water bottles. Blowing high‐humidity air onto the surface of the sensor can raise the ambient humidity of the sensor and the small space around it. Using the 3D printing, shims were prepared with the same height and different curvatures (Figure S13c, Supporting Information). The shims are bonded to the indenter of the device, and the sensors are attached to the surface of the shims.

The experiments were performed on the responses of a pressure sensor array to the regularly varying 3D force. The 3D force was applied to the sensor array by moving the manipulator clamping a push‐pull gauge in a predefined direction, meanwhile the force was recorded. The pressure amplitude was proportional to the displacement of the manipulator in the pressing direction. When the sensor array was placed directly under the indenter of the push‐pull gauge, the manipulator can provide the sensor with a force of any direction and amplitude in positive normal vector space (Figure S30, Supporting Information).

### Algorithmic Architecture

The pressure and triboelectricity data were fed into a fully connected network for alignment and then stitched together and fed into a Transformer Encoder for feature extraction, which used the original Encoder module. The data was fed into the multi‐head attention module for computation and extraction of features from the data through the Add & Norm and Feed Forward layers. Finally, the features obtained by the transformer encoder were fed into a classification prediction head for prediction. The prediction head used a multilayer perceptron (MLP).

The datasets collected by volunteer #1 were used for training to get the weights of the model. Migration learning was then performed using a small number of datasets collected by volunteers #2 and #3. The datasets sampled by Volunteer #2 and #3 had only 20% of the data volume of Volunteer #1. The model parameters of the Transformer Encoder were frozen for the migration learning. The new dataset was used to train the data alignment module and the prediction head MLP.

## Conflict of Interest

The authors declare no conflict of interest.

## Supporting information

Supporting Information

Supplemental Movie 1

## Data Availability

The data that support the findings of this study are available from the corresponding author upon reasonable request.
