# Supplementary material for: A Multifunctional Tactile Sensory System for Robotic Intelligent Identification and Manipulation Perception
Source: Adv Sci (Weinh). 2024 Sep 9;11(41):2402705. doi: 10.1002/advs.202402705 (PMC11538698; doi:10.1002/advs.202402705)
Supplement: Supplementary file 1 — Supporting Information [file ADVS-11-2402705-s002.docx]

**A Multifunctional Tactile Sensory System for Robotic Intelligent Identification and Manipulation Perception**

*Yue Jiang, Lin Fan,* *Xilong Sun, Zehe Luo, Herong Wang, Rucong Lai, Jie Wang, Qiyang Gan, Ning Li,* * *and Jindong Tian* *

L. Fan, H. Wang, J. Wang, J. Tian

Key Laboratory of Optoelectronic Devices and Systems of Ministry of Education and Guangdong Province, College of Physics and Optoelectronic Engineering, Shenzhen University, Shenzhen 518060, China

Guangdong Laboratory of Artificial Intelligence and Digital Economy (Shenzhen), Shenzhen University, Shenzhen 518132, China

Email: [jindt@szu.edu.cn](mailto:jindt@szu.edu.cn)

X. Sun, Z. Luo, Q. Gan, N. Li,

Guangdong Laboratory of Artificial Intelligence and Digital Economy (Shenzhen), Shenzhen University, Shenzhen 518132, China

Email: ln5858518@hotmail.com,

Y. Jiang

Key Laboratory of Optoelectronic Devices and Systems of Ministry of Education and Guangdong Province, College of Physics and Optoelectronic Engineering, Shenzhen University, Shenzhen 518060, China

College of Computer Science and Software Engineering, Shenzhen University, Shenzhen 518060, China

Guangdong Laboratory of Artificial Intelligence and Digital Economy (Shenzhen), Shenzhen University, Shenzhen 518132, China

Rucong Lai

Institute of Applied Physics and Materials Engineering, University of Macau, Macao 999078, China


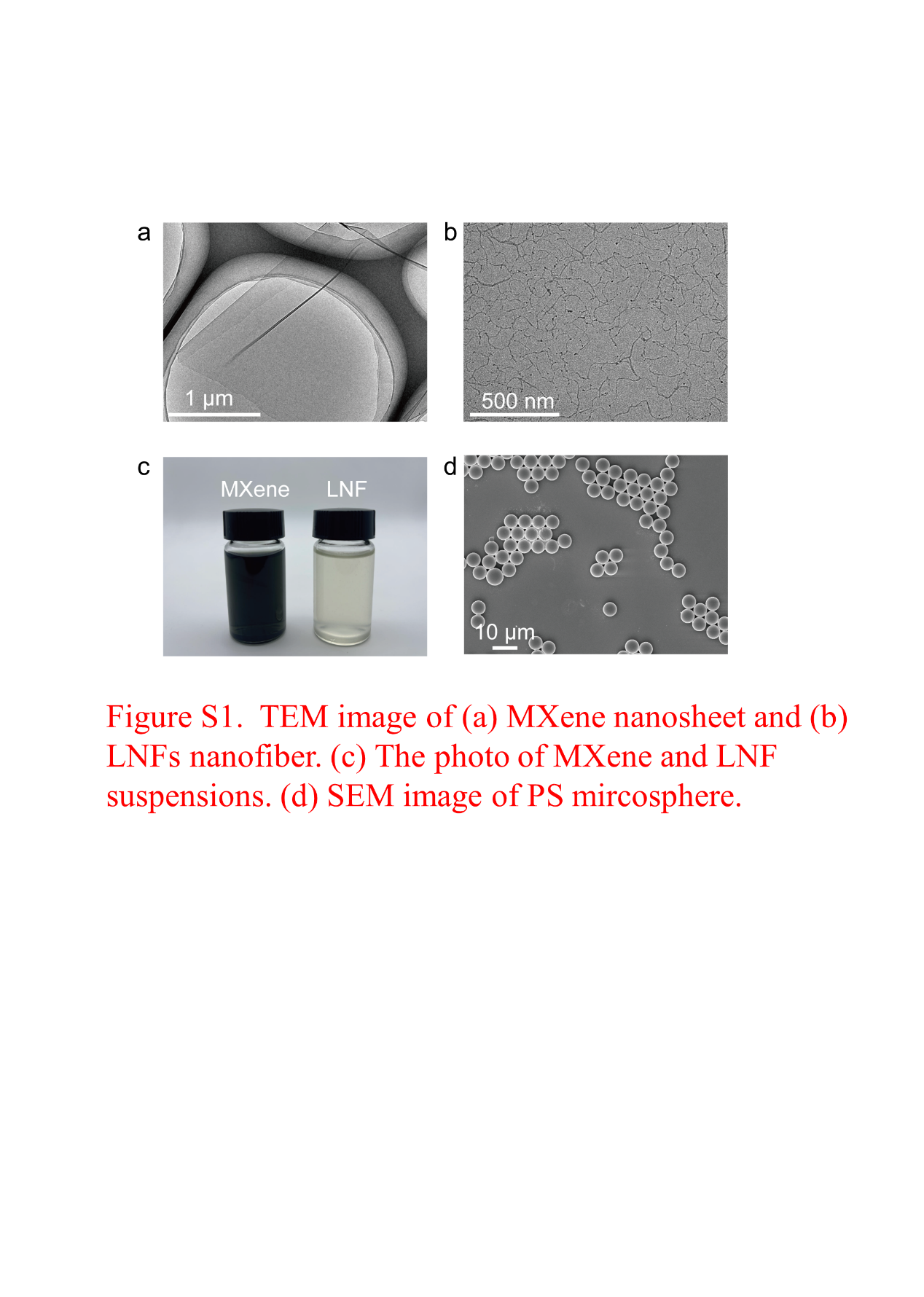


Figure S1. (a,b) TEM images of (a) MXene nanosheet and (b) LNF nanofibers. (c) The photo of MXene and LNF suspensions. (d) SEM image of PS microspheres.


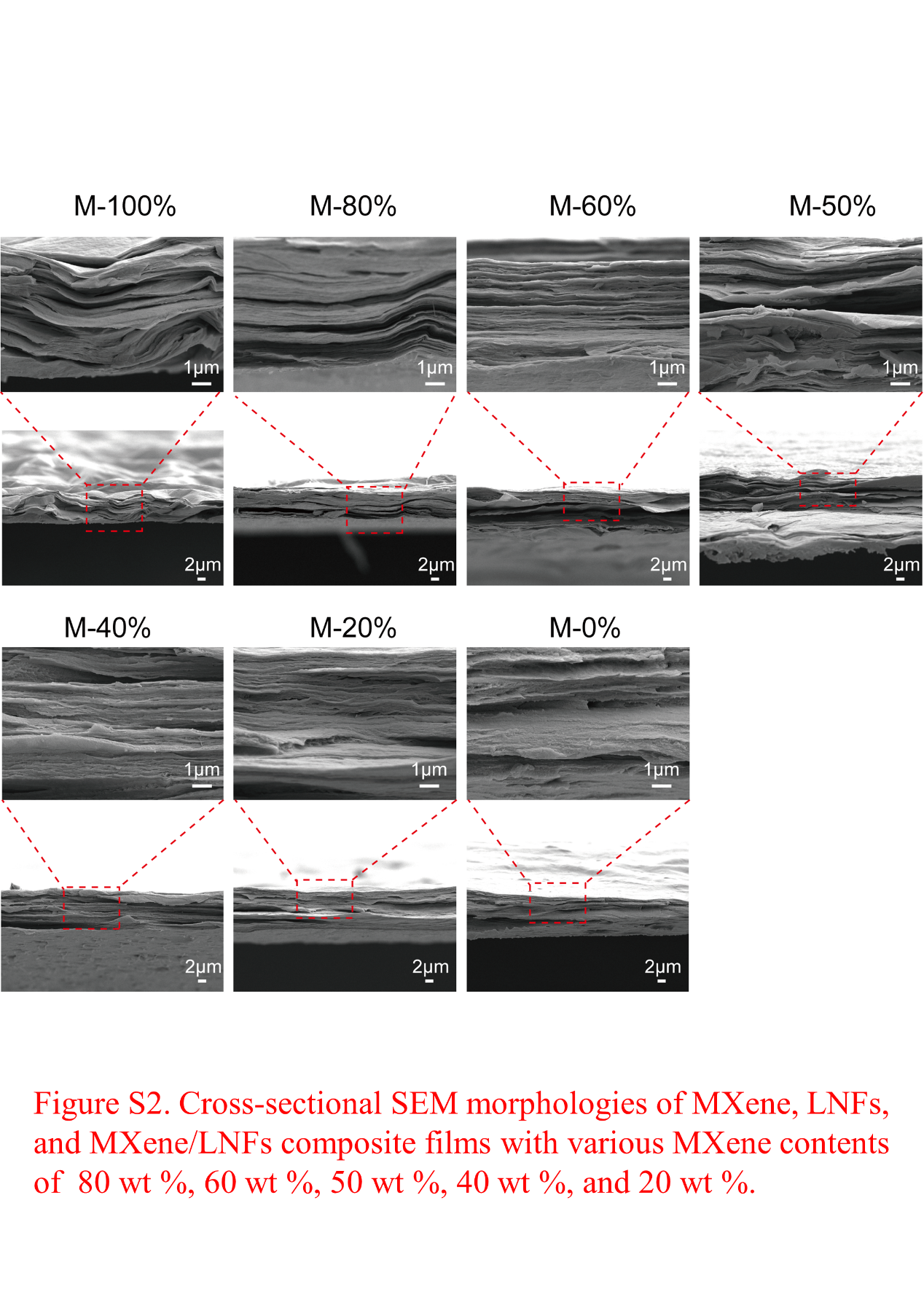


Figure S2. SEM images of cross-sectional morphologies of MXene, LNF, and MXene/LNF composite films with various MXene contents of 80 wt%, 60 wt%, 50 wt%, 40 wt%, and 20 wt%.


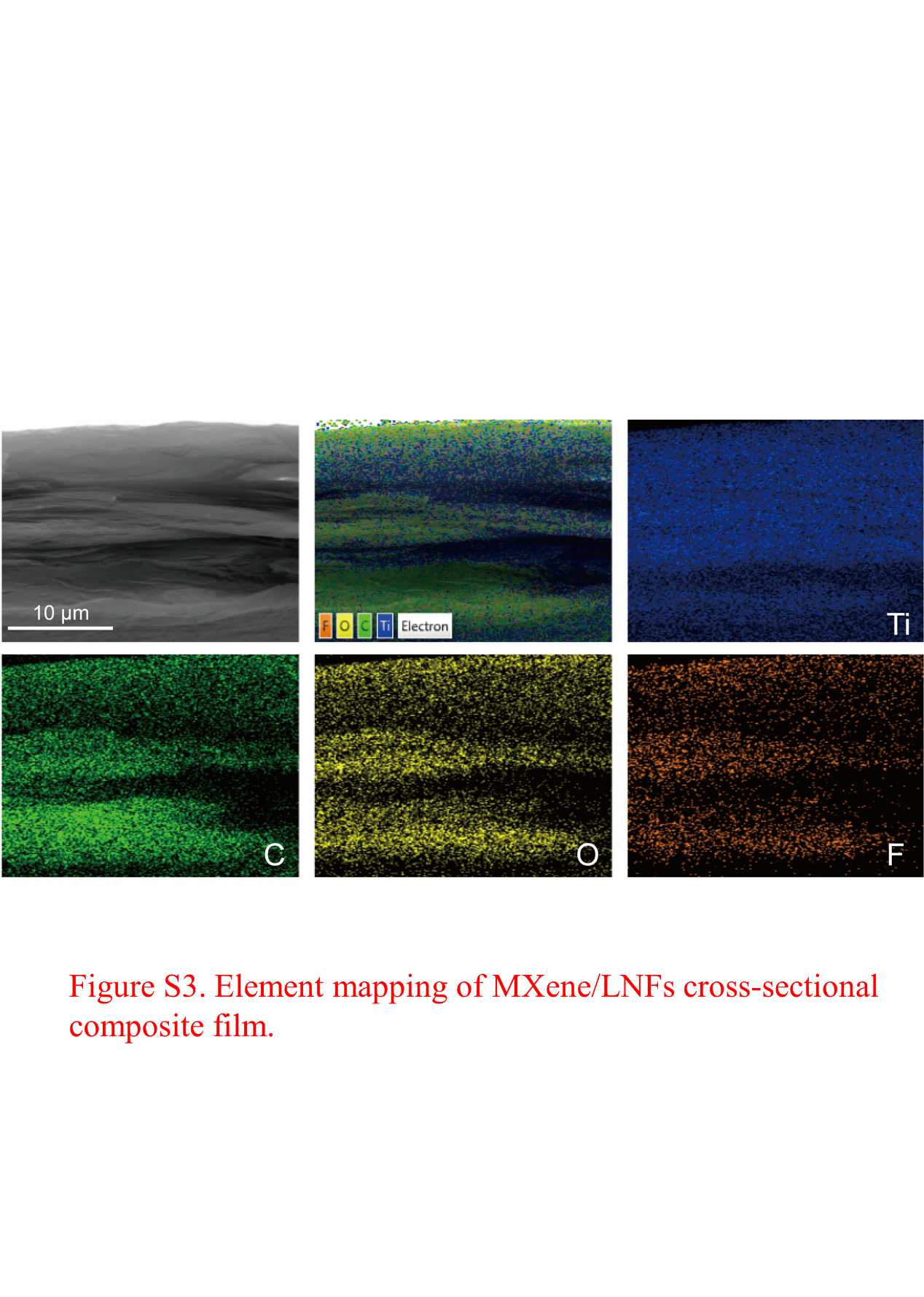


Figure S3. Element mapping of MXene/LNF composite film.


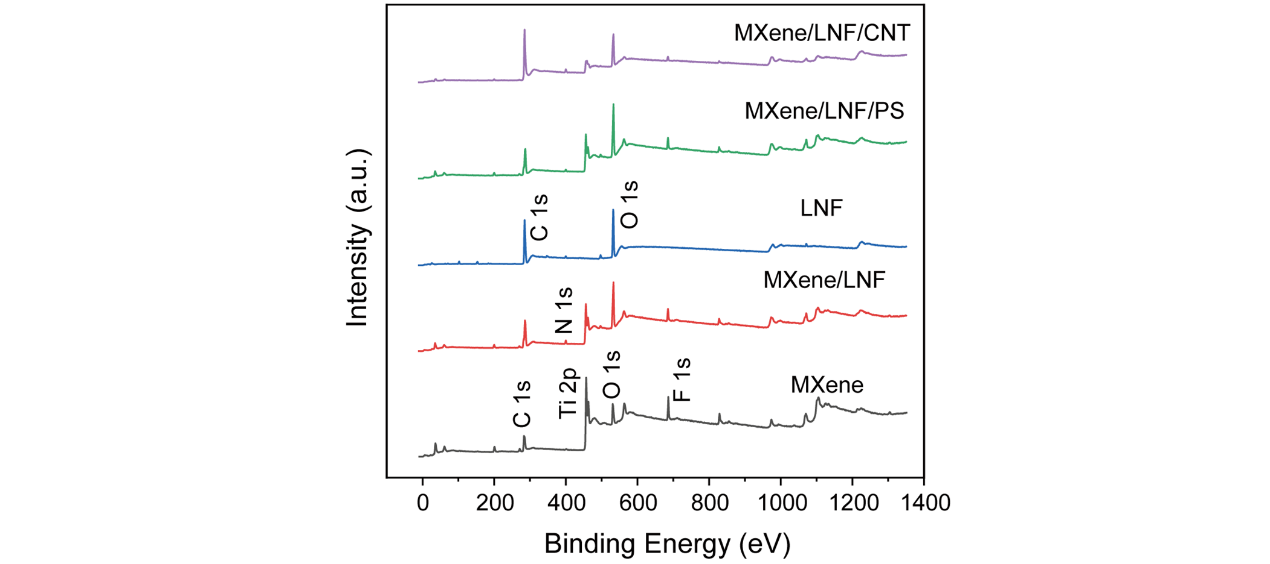


Figure S4. XPS survey spectrums of MXene, LNF, MXene/LNF, MXene/LNF/PS, and MXene/LNF/CNT composite films.


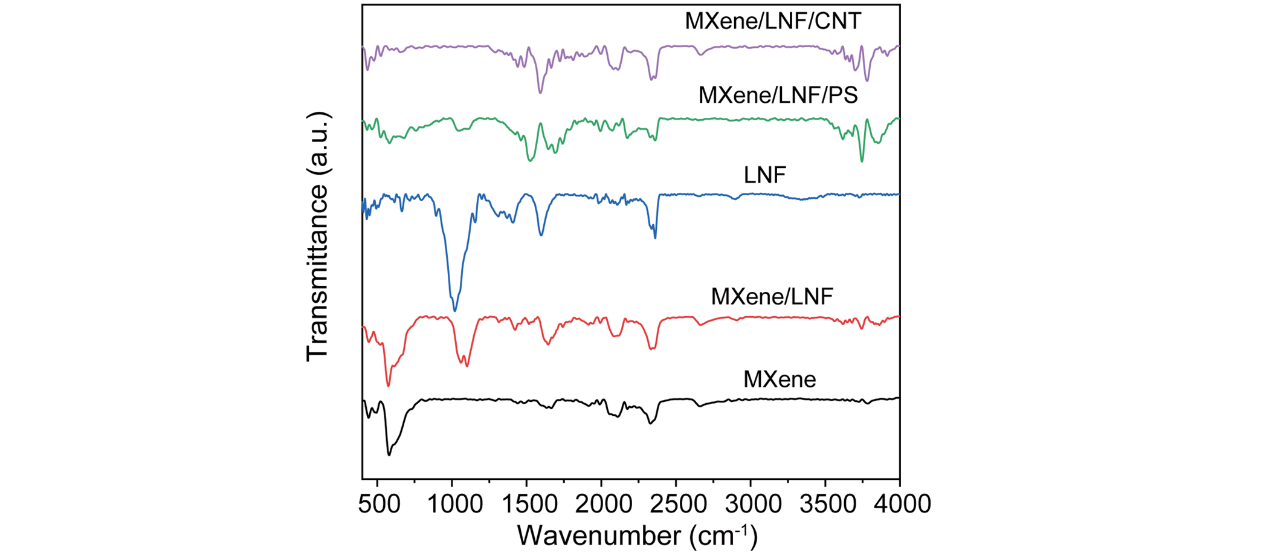


Figure S5. FTIR spectra of the MXene, LNF, MXene/LNF, MXene/LNF/PS, and MXene/LNF/CNT composite films.


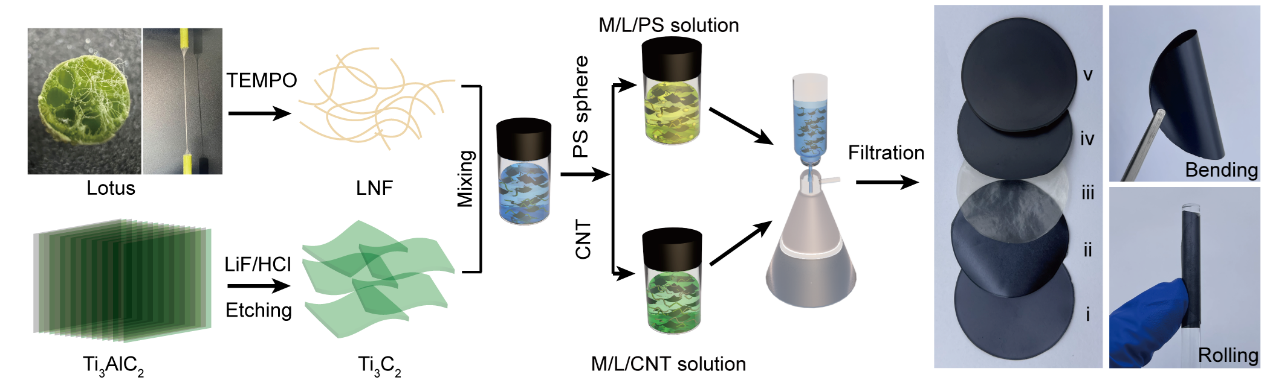
Figure S6. Schematic illustration for the preparation of the films, and the digital photographs of the prepared films: (ⅰ) MXene, (ⅱ) MXene/LNF, (ⅲ) LNF, (ⅳ) MXene/LNF/PS, (ⅴ) MXene/LNF/CNT.


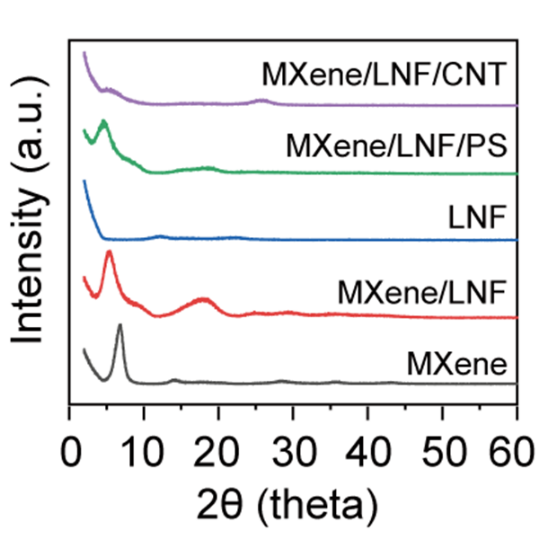


Figure S7. XRD patterns of the diverse functional films. Typical tensile stress versus strain curves.


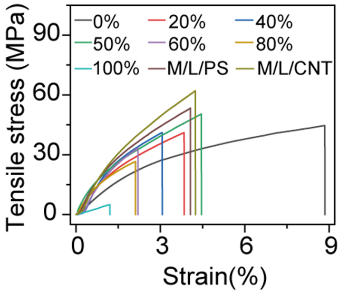


Figure S8. Typical tensile stress versus strain curves of the films.


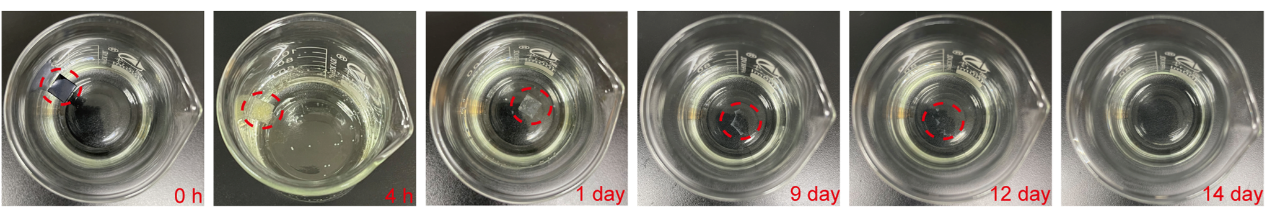


Figure S9. The complete-degradation process of a MXene/LNF film in 1 wt% H_2_O_2_ solution.


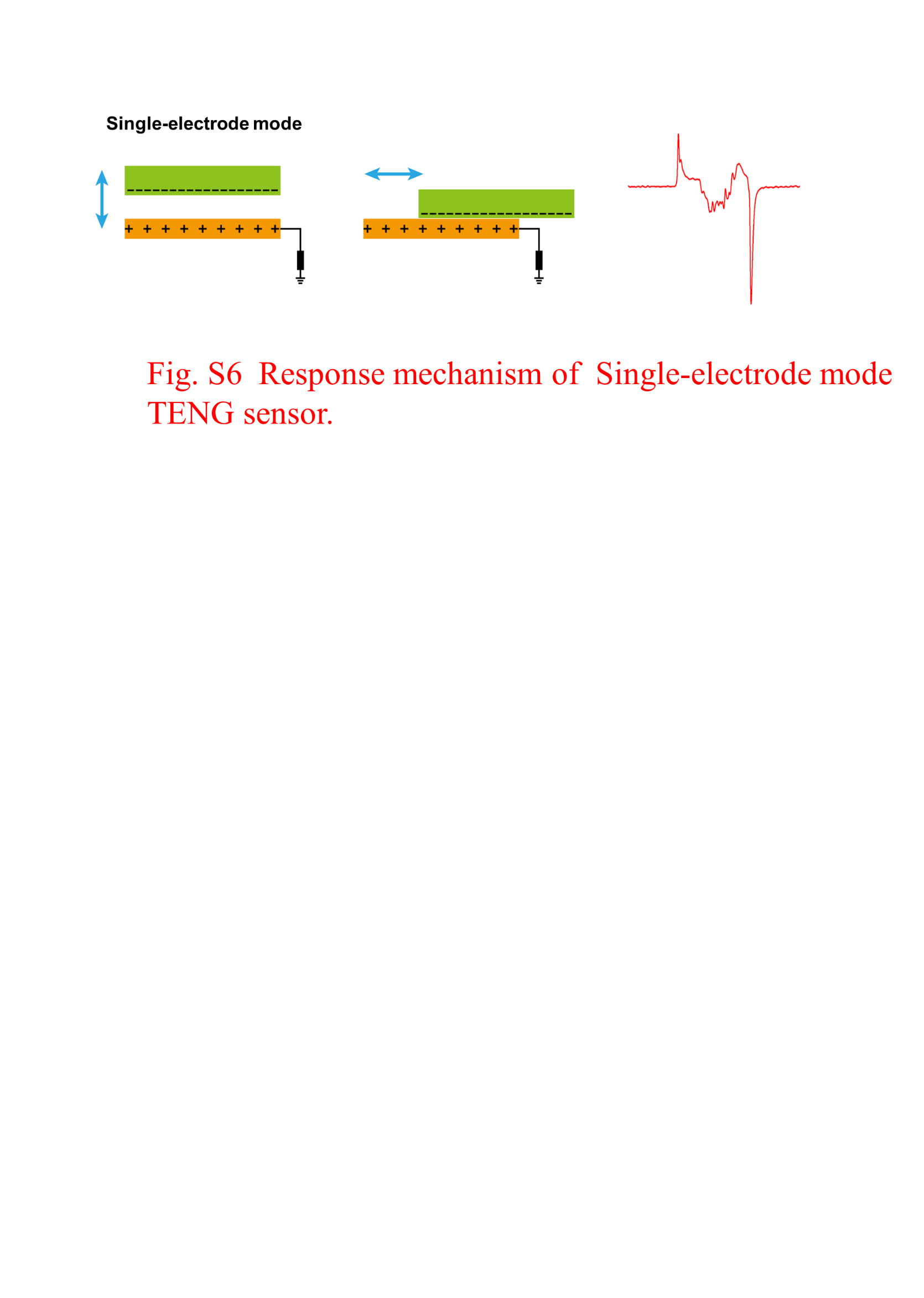


Figure S10. Response mechanism of the triboelectric sensor.


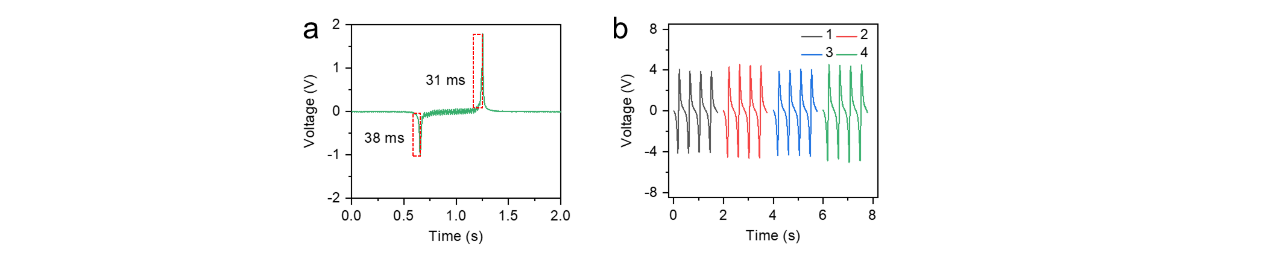


Figure S11. (a) Response time (38 ms) and recovery time (31 ms) of MXene/LNF/CNT film-based TENG sensor. (b) the consistency of the TENG sensor.


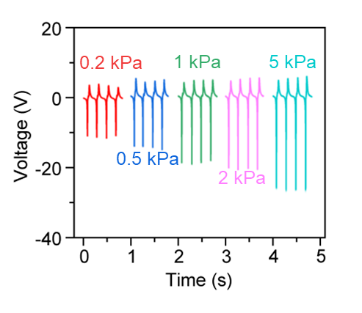


Figure S12. The output voltage of the TENG responding to the changes in the different applied pressures.


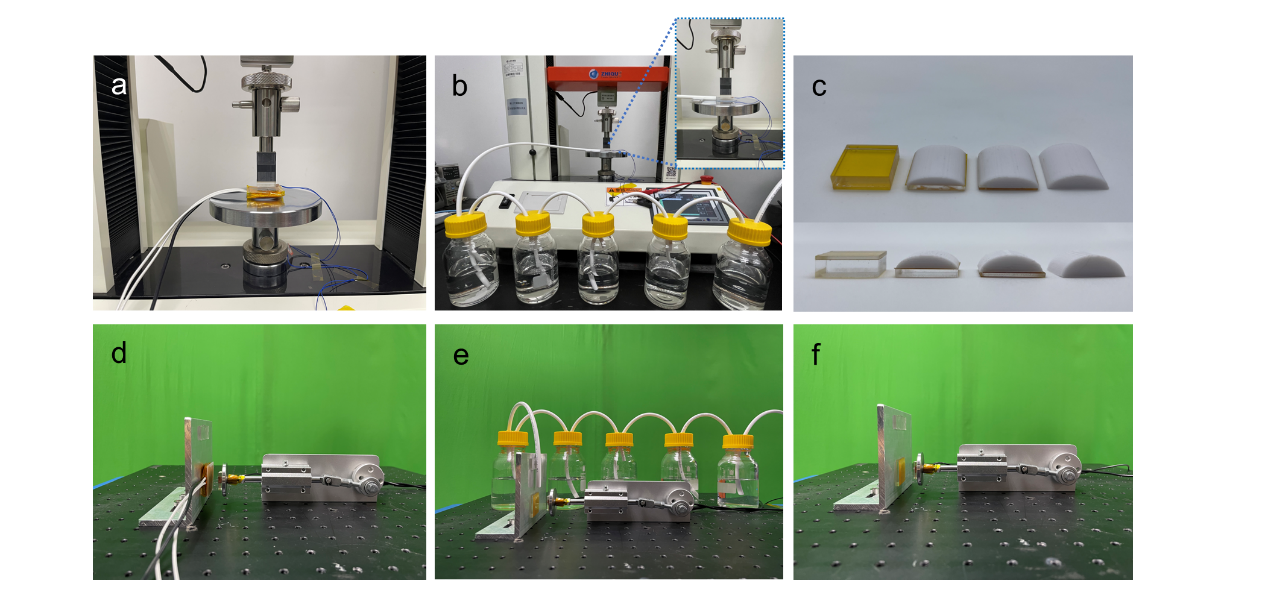


Figure S13. (a, b) Experimental setup for the measuring of the responses of pressure sensors to the applied pressure at (a) different temperatures and (b) environment humidities, respectively. (c) photograph of the molds used to provide different deformation states for sensors. (d, e) Experimental setup for the measuring of the responses of TENG and capacitive sensor to the applied pressure at (d) different temperatures and (e) different humidities, respectively. (f) Experimental setup for the measuring of the responses of pressure sensors to the applied pressure under different deformation states, where the molds are attached to the surface of Al sheet.


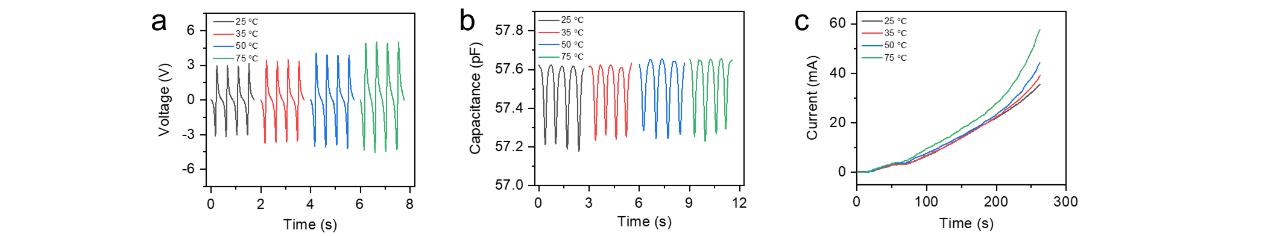


Figure S14. Responses of the (a)TENG, (b) capacitive sensor and (c) pressure sensor to the applied pressures at different temperatures.


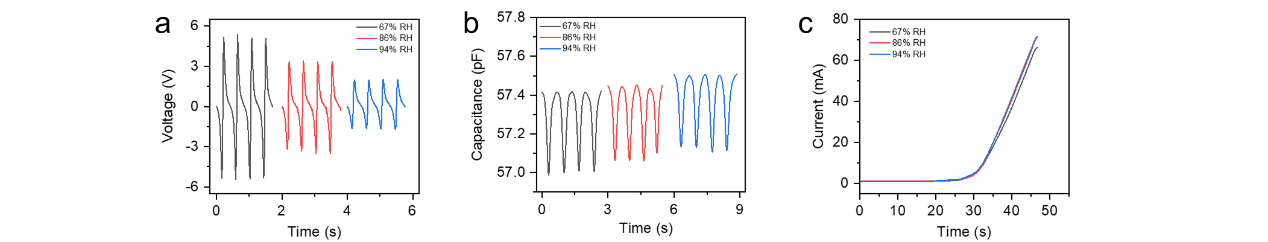


Figure S15. Responses of the (a)TENG, (b) capacitive sensor and (c) pressure sensor to the applied pressures at different environment humidities.


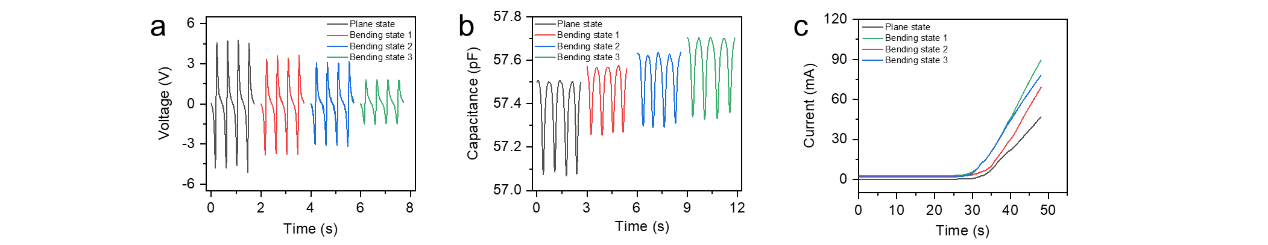


Figure S16. Responses of the (a)TENG, (b) capacitive sensor and (c) pressure sensor to the applied pressures under the plane state and deformation states.


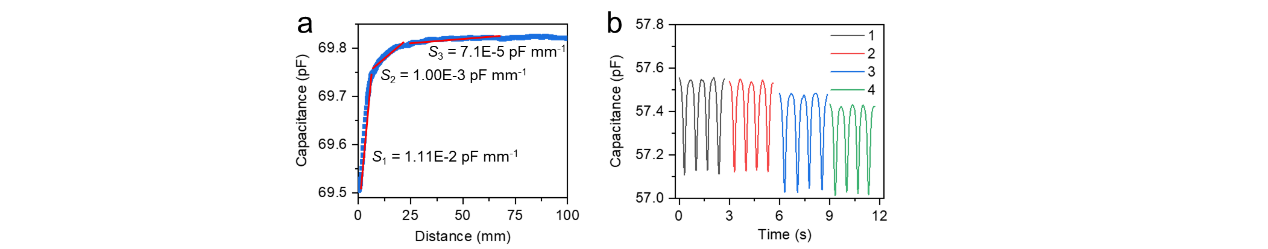


Figure S17. (a) The sensitivity and (b) consistency of MXene/LNF/CNT film-based capicitive sensor.


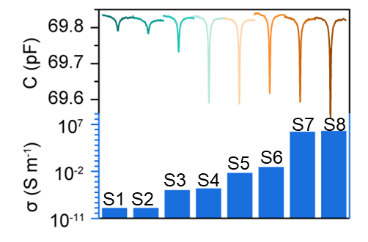


Figure S18. The response of the capacitive sensor when approaching, contacting and leaving the targets with different conductivities at a fixed speed from a distance of 5 cm. (S1: Plastic cup, S2: Disposable cup, S3: Ethyl alcohol, S4: Pure water, S5: Sucrose solution, S6: Salt solution, S7: Aluminum, S8: Copper)


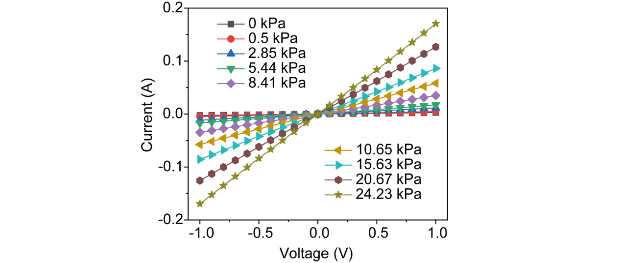


Figure S19. *I*–*V* curves of the pressure sensor applied with various pressures.


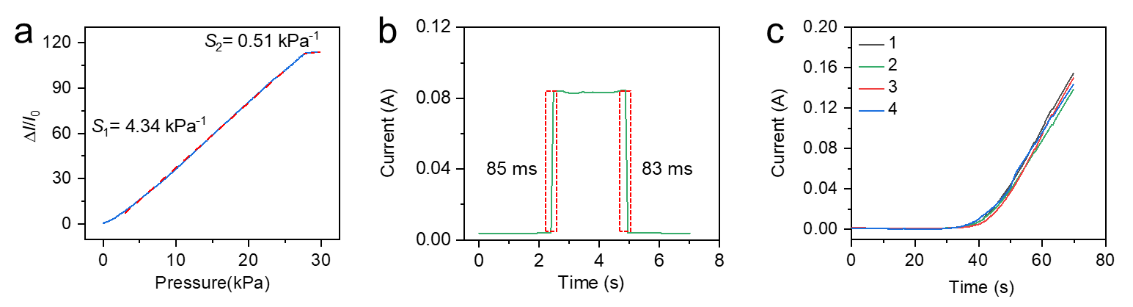


Figure S20. (a) The sensitivity (4.34 kPa^-1^), (b) Response time (85 ms) and recovery time (83 ms) and (c) consistency of MXene/LNF/PS film-based pressure sensor.


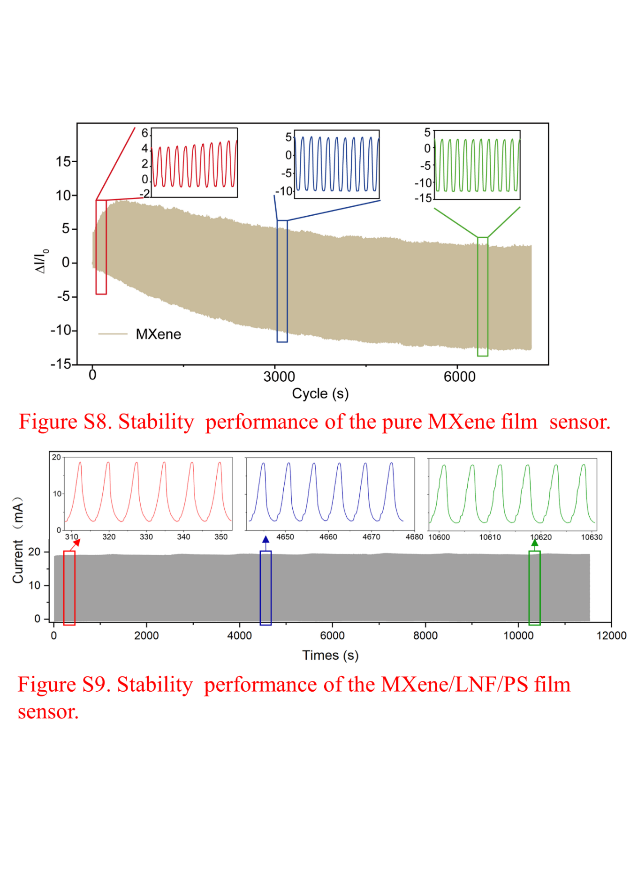


Figure S21. Stability performance of the pure MXene film-based pressure sensor.


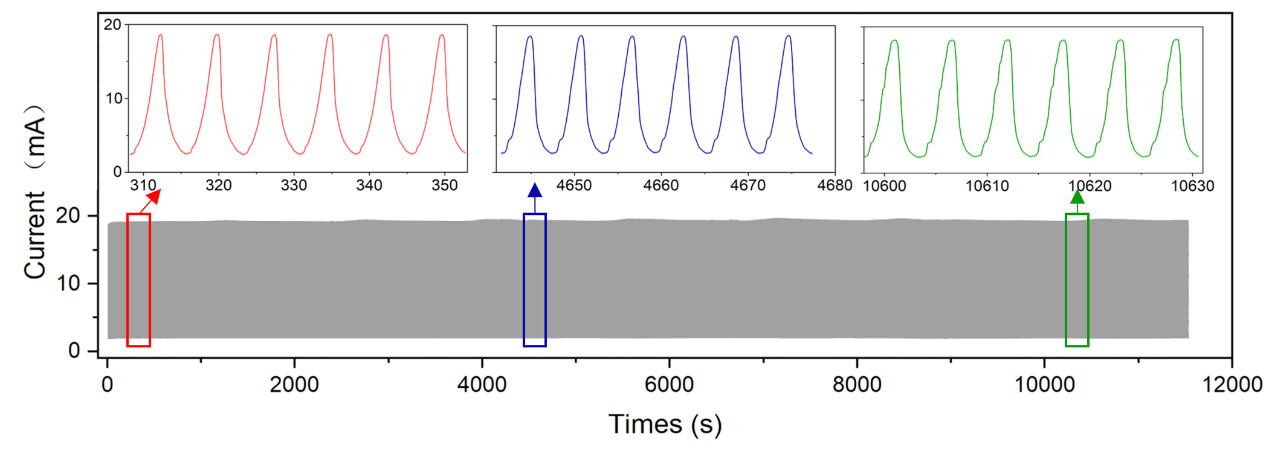


Figure S22. Stability performance of the MXene/LNF/PS film-based pressure sensor.


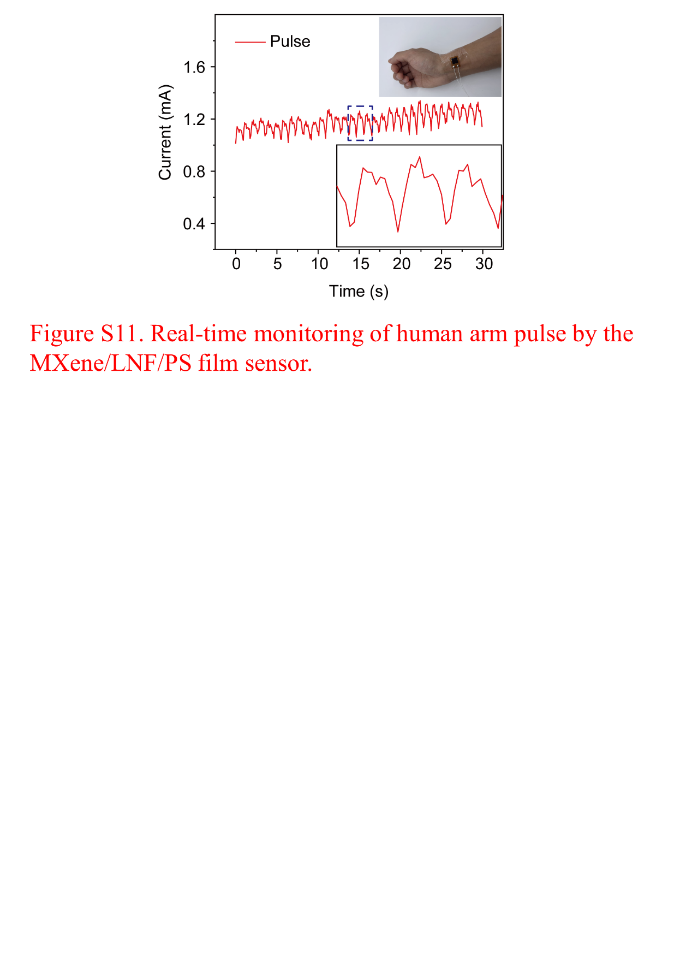


Figure S23. Real-time monitoring of human arm pulse by the MXene/LNF/PS film-based pressure sensor.


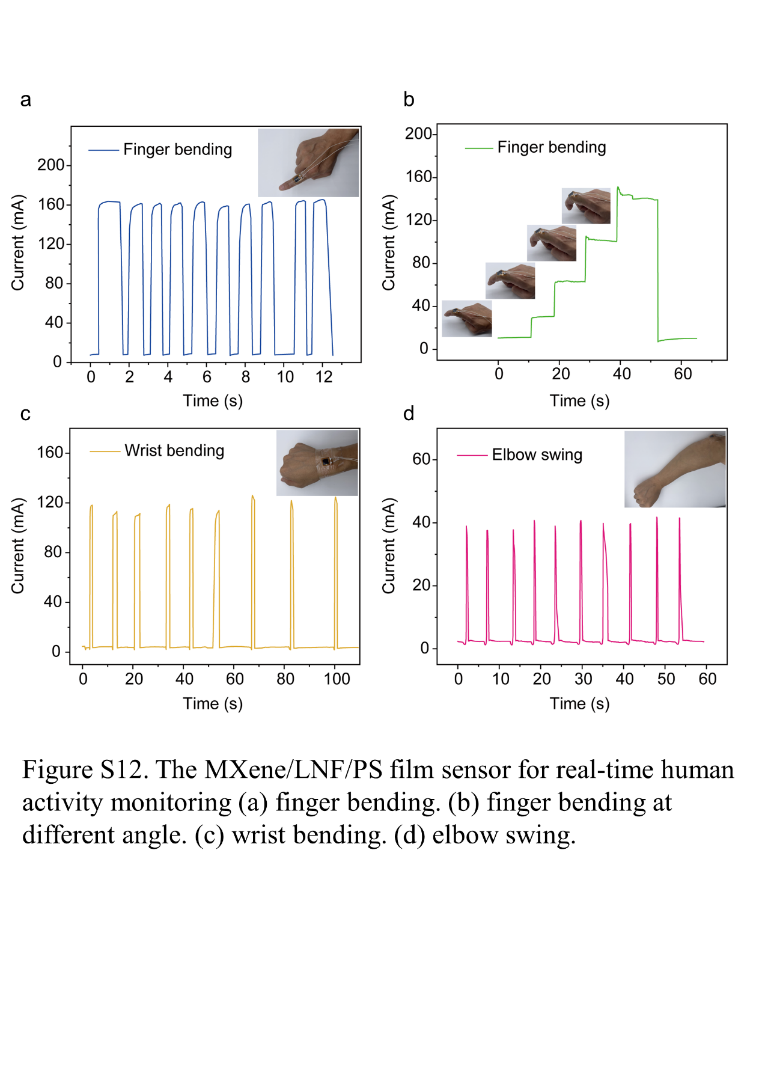


Figure S24. The MXene/LNF/PS film-based pressure sensor for the real-time monitoring of human activity: (a) cyclic finger bending, (b) finger bending at different angles, (c) cyclic wrist bending and (d) cyclic elbow swing.


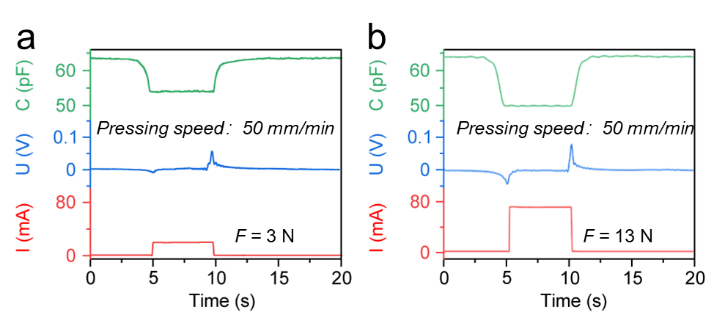


Figure S25. The sensing signals of the multi-parameter sensor responding to light pressing (a) and heavy pressing (b) actions that differ in pressure and approaching speed.


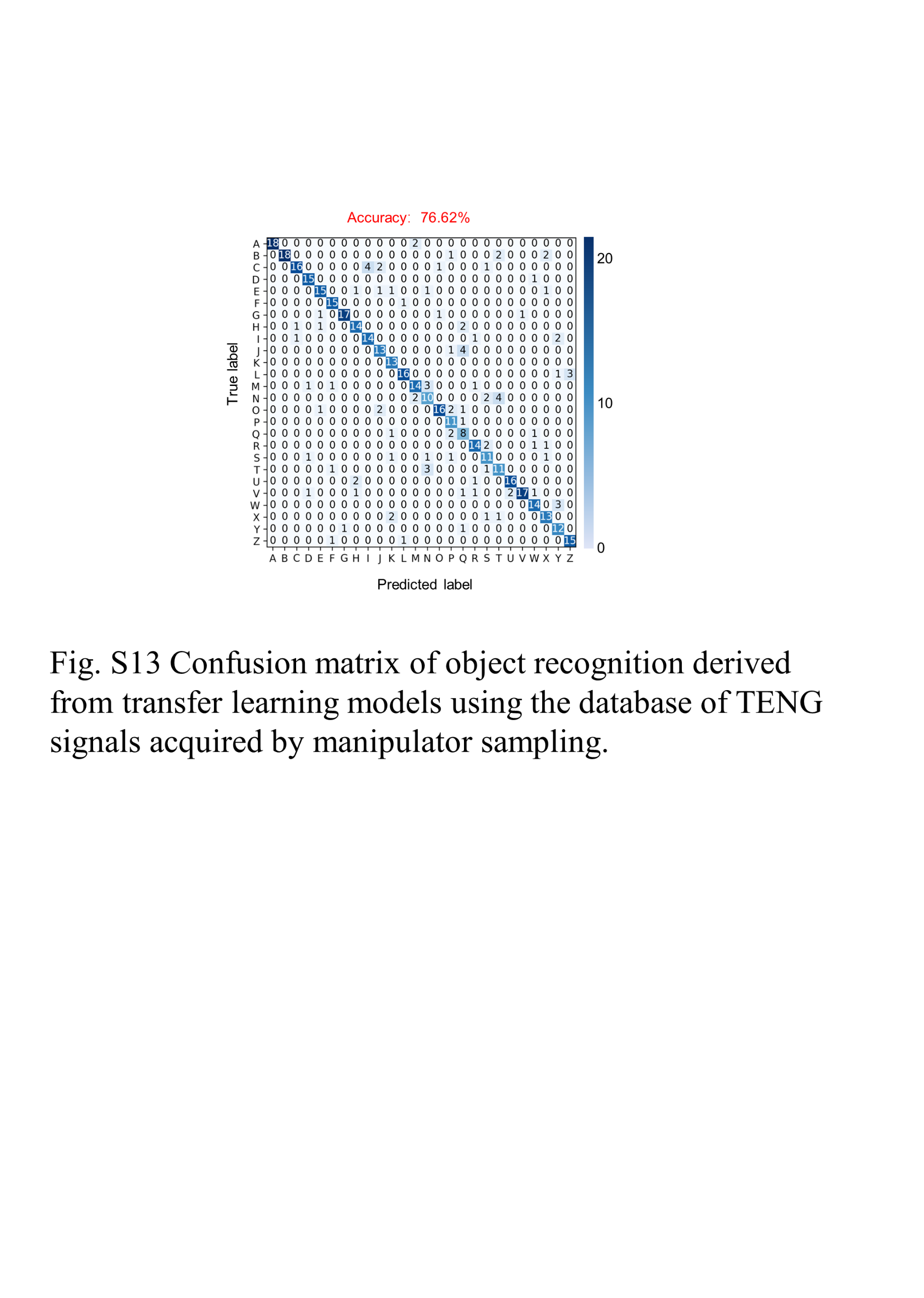


Figure S26. Confusion matrix of object recognition derived from transfer learning model using the database of triboelectric signals acquired by manipulator sampling.


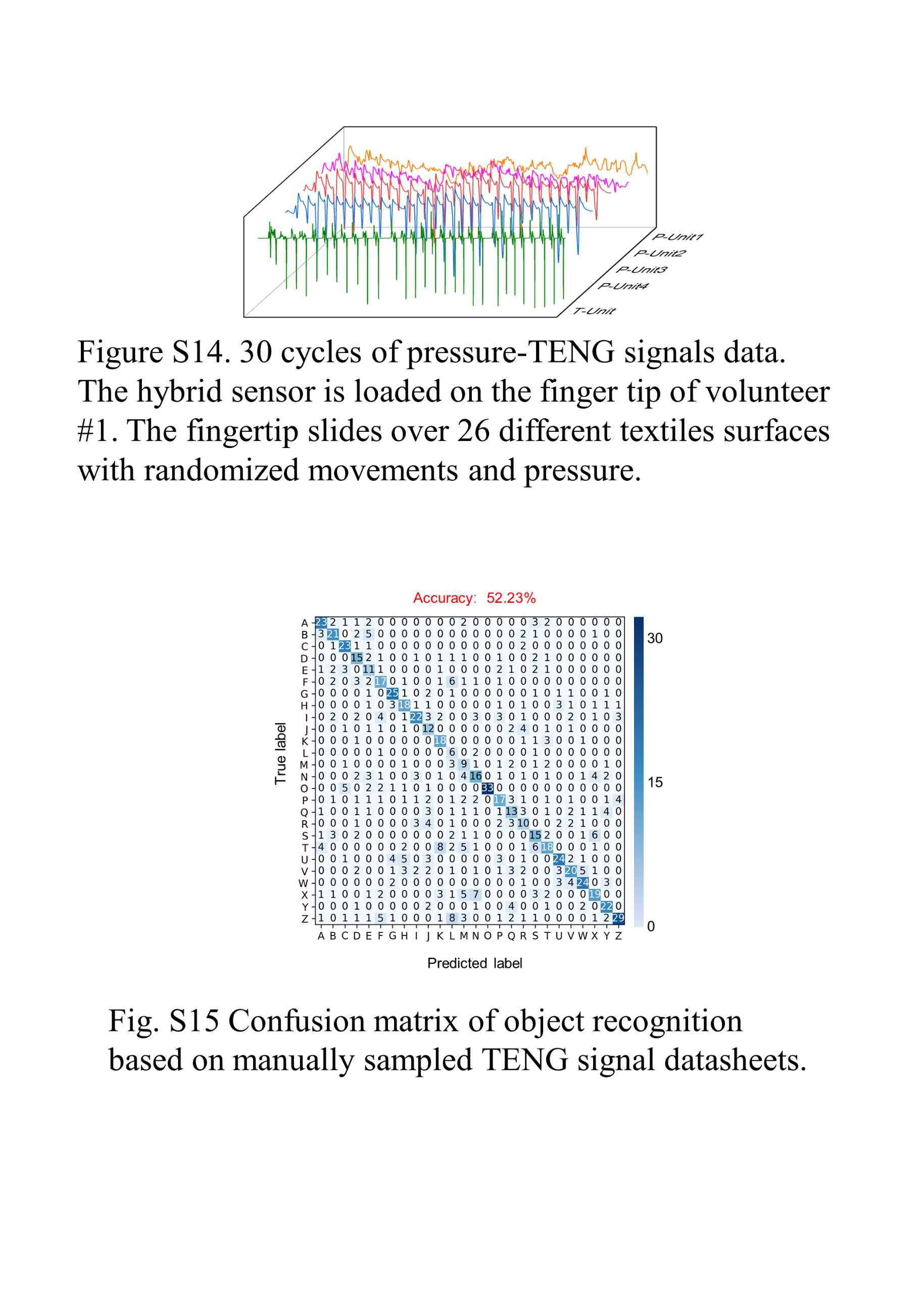


Figure S27. 30 cycles of pressure-triboelectricity signals data. The dual-modal sensor is loaded on the fingertip of volunteer #1. The fingertip slides over the surface of 26 textiles with randomized movements and pressure.


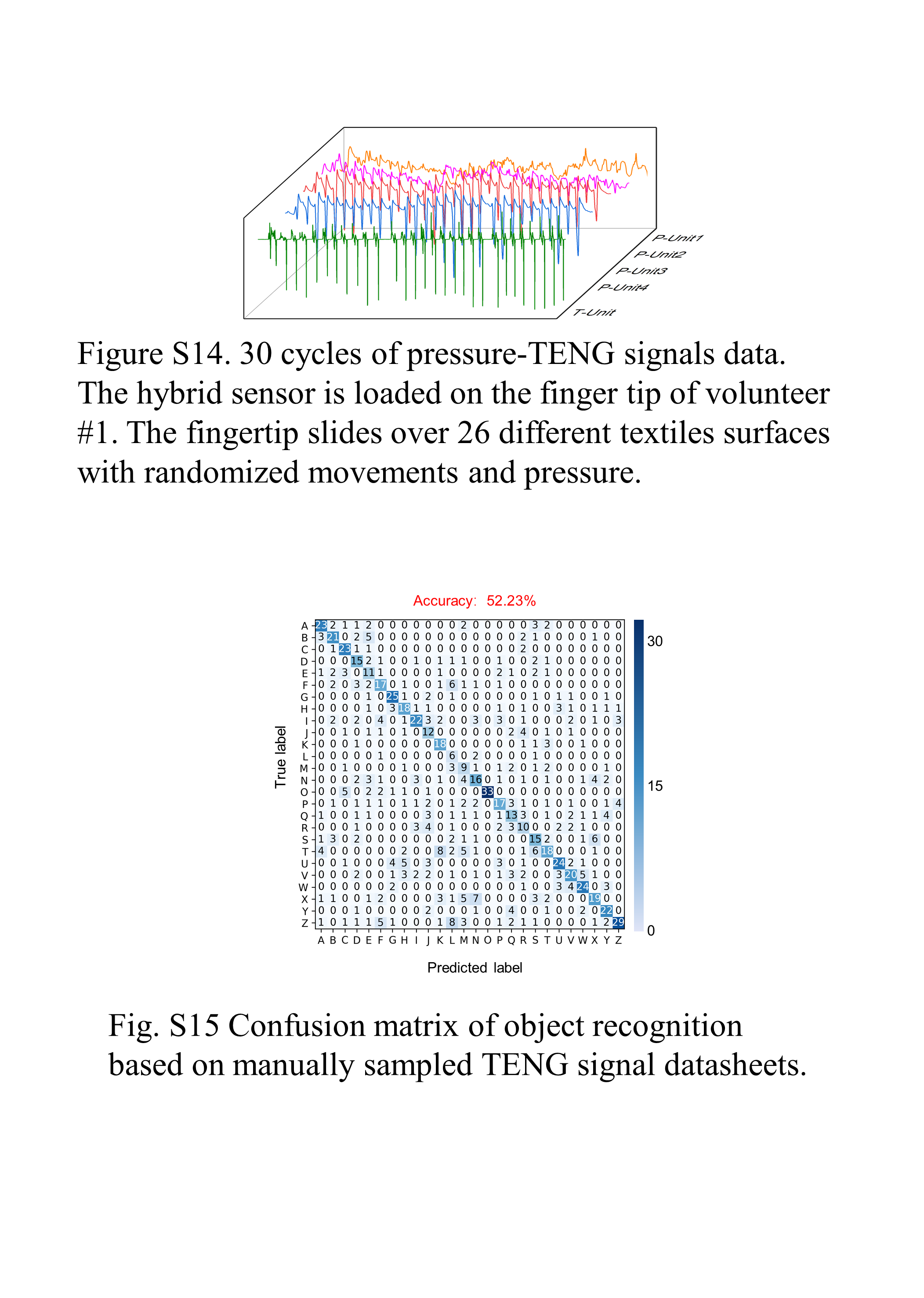


Figure S28. Confusion matrix of object recognition based on manually sampled triboelectric signal datasets.


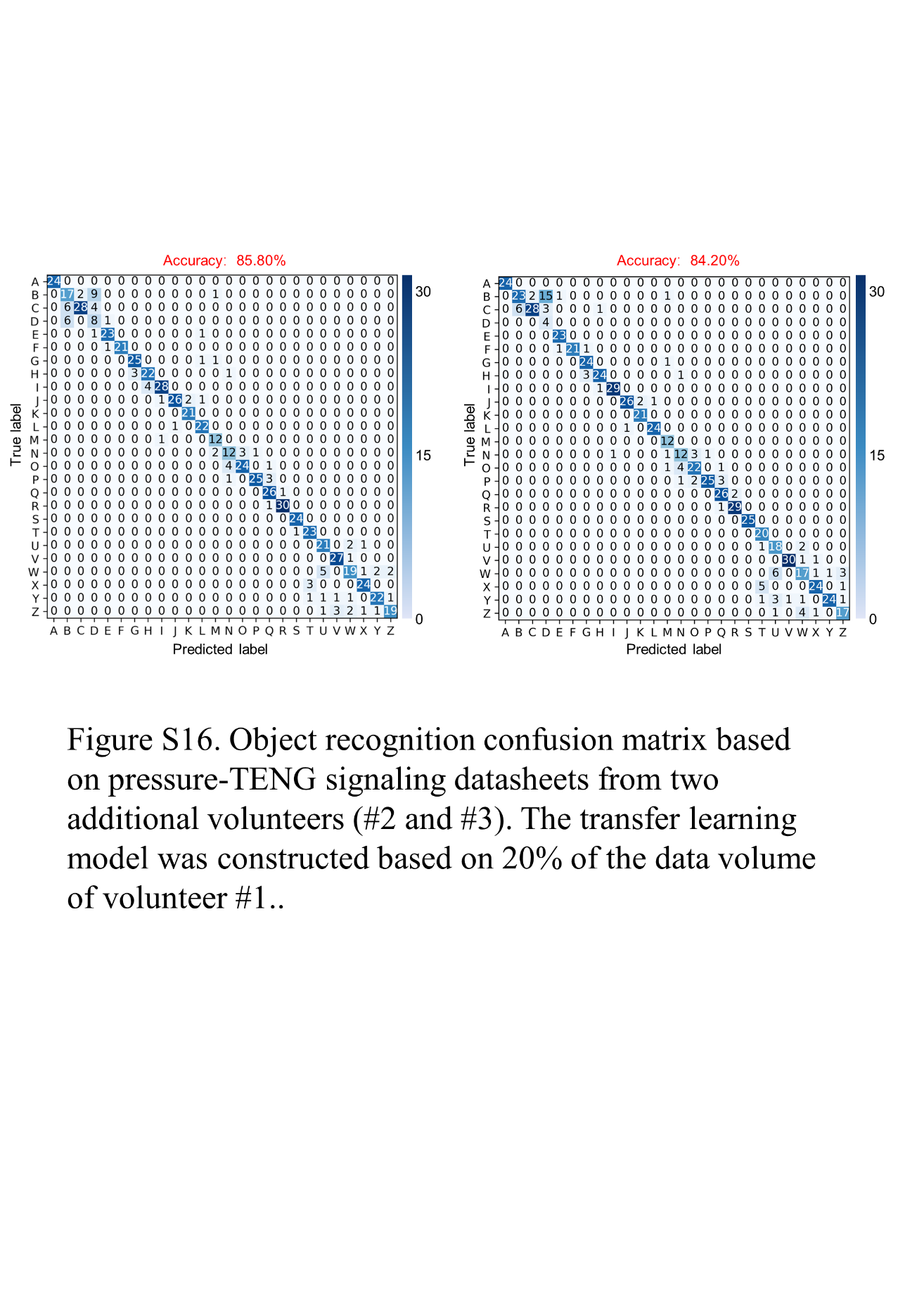


Figure S29. Confusion matrix of object recognition based on pressure-triboelectric signal datasets from two volunteers (#2 and #3). The transfer learning model is constructed based on 20% of the datasets of volunteer #1.


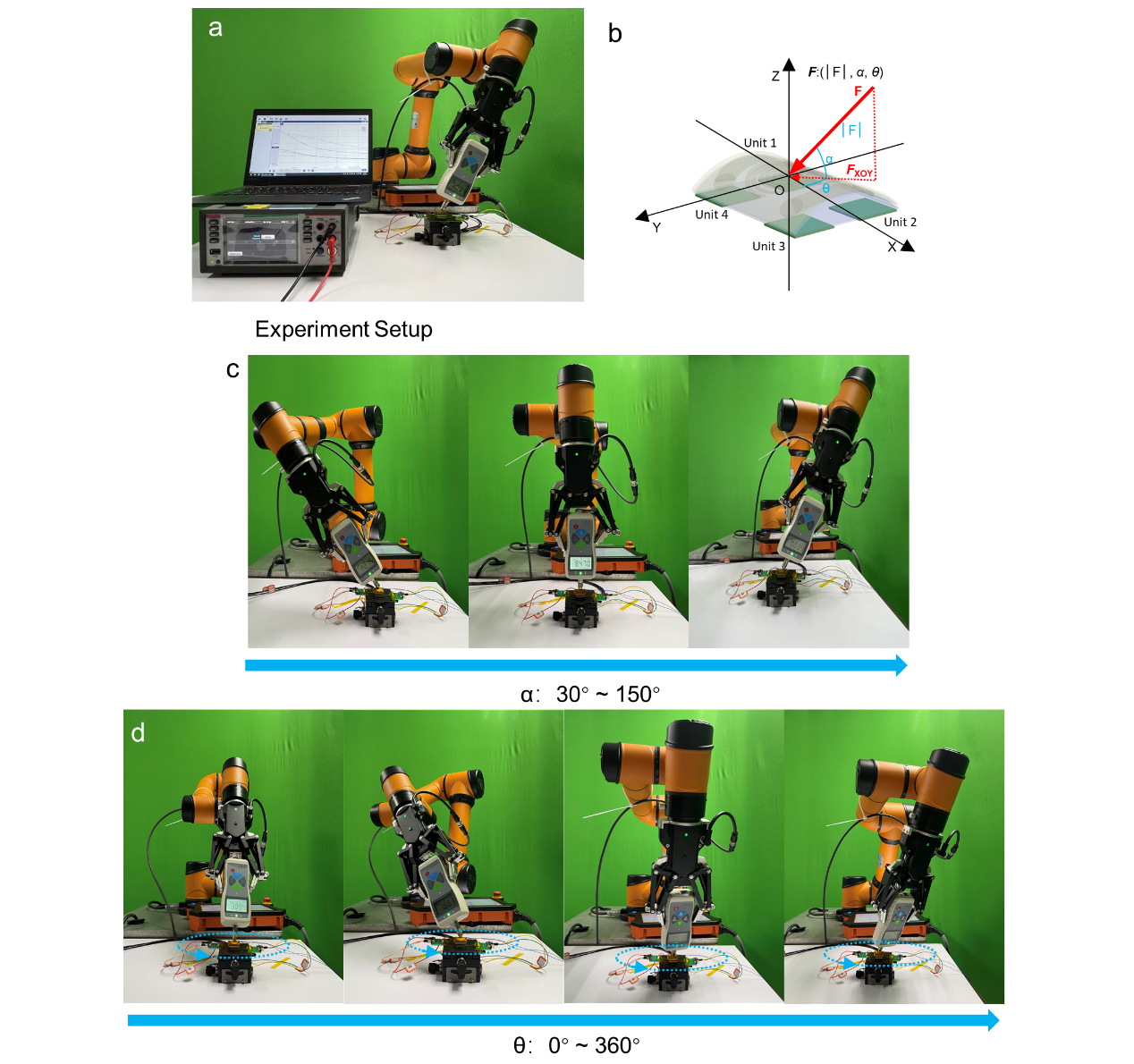


Figure S30 (a) The experiments setup of pressure sensor arrays to the regularly varying three-dimensional force. (b) Parametric decomposition of three dimensions force. (c, d) Regularly varying three-dimensional force with a single variable (α or θ).


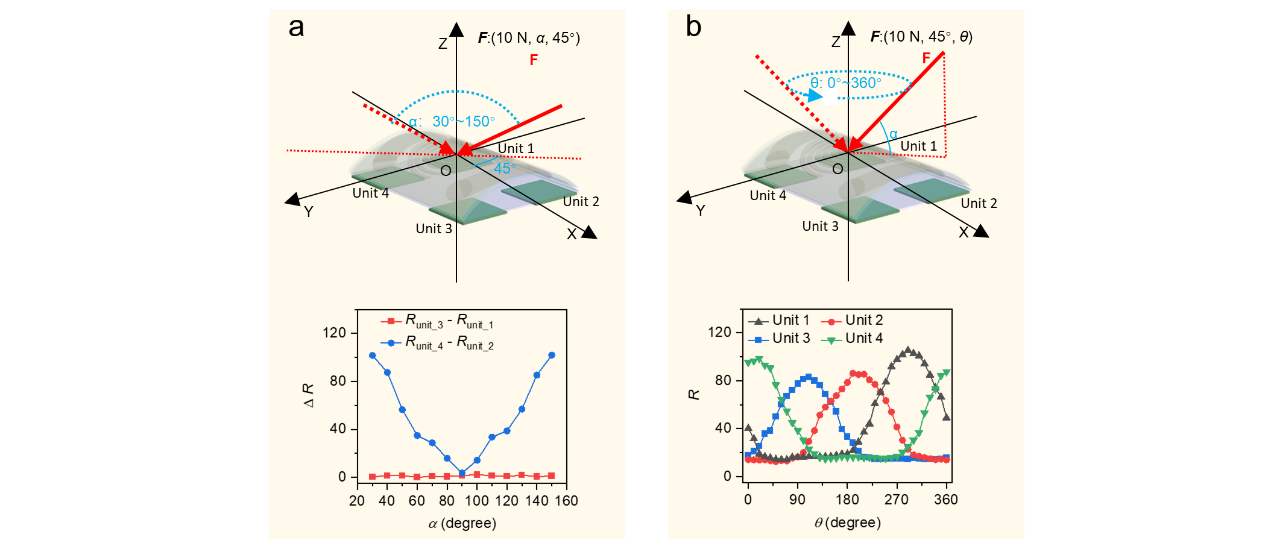


Figure S31 (a) The difference in the resistance responses of the four pressure units, when f = 10 N, θ = 45° and α is from 30° to 150°. (b) The resistance responses of the four pressure units, when f = 10 N, α = 45° and θ is from 0° to 360°.


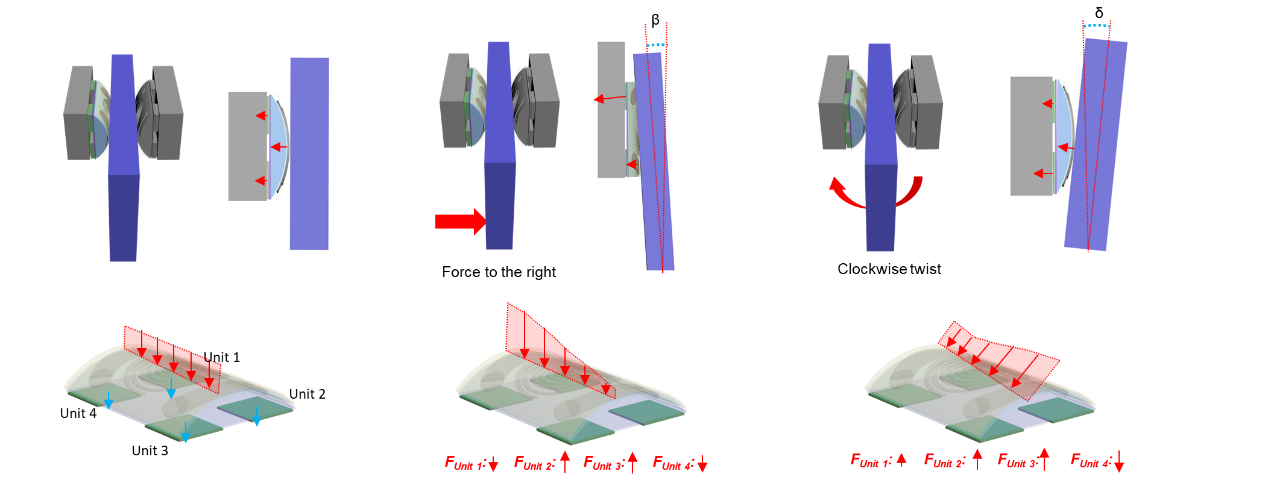


Figure S32 Force variations of the sensor array under different stress states of the plastic plate.


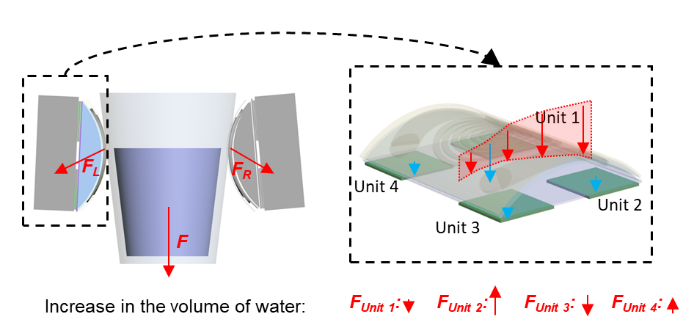


Figure S33 Force variations of the sensor array as the amount of water in the cup increases.


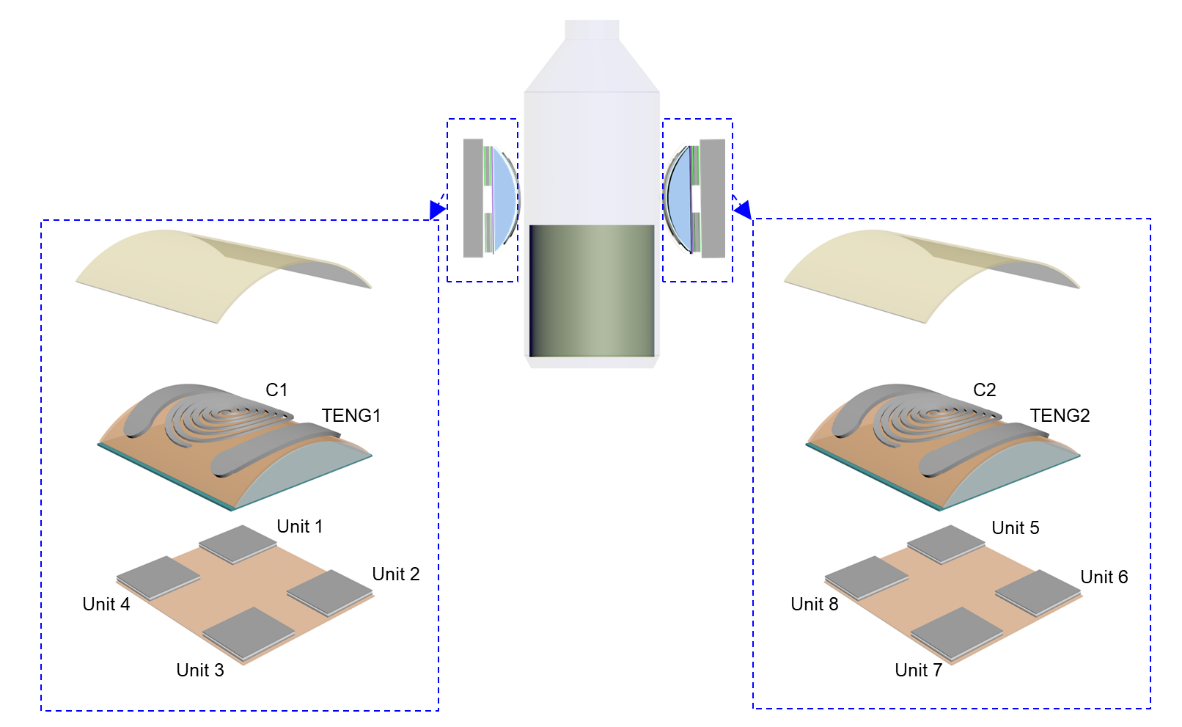


Figure S34. The loading position of the multimodal tactile sensors.


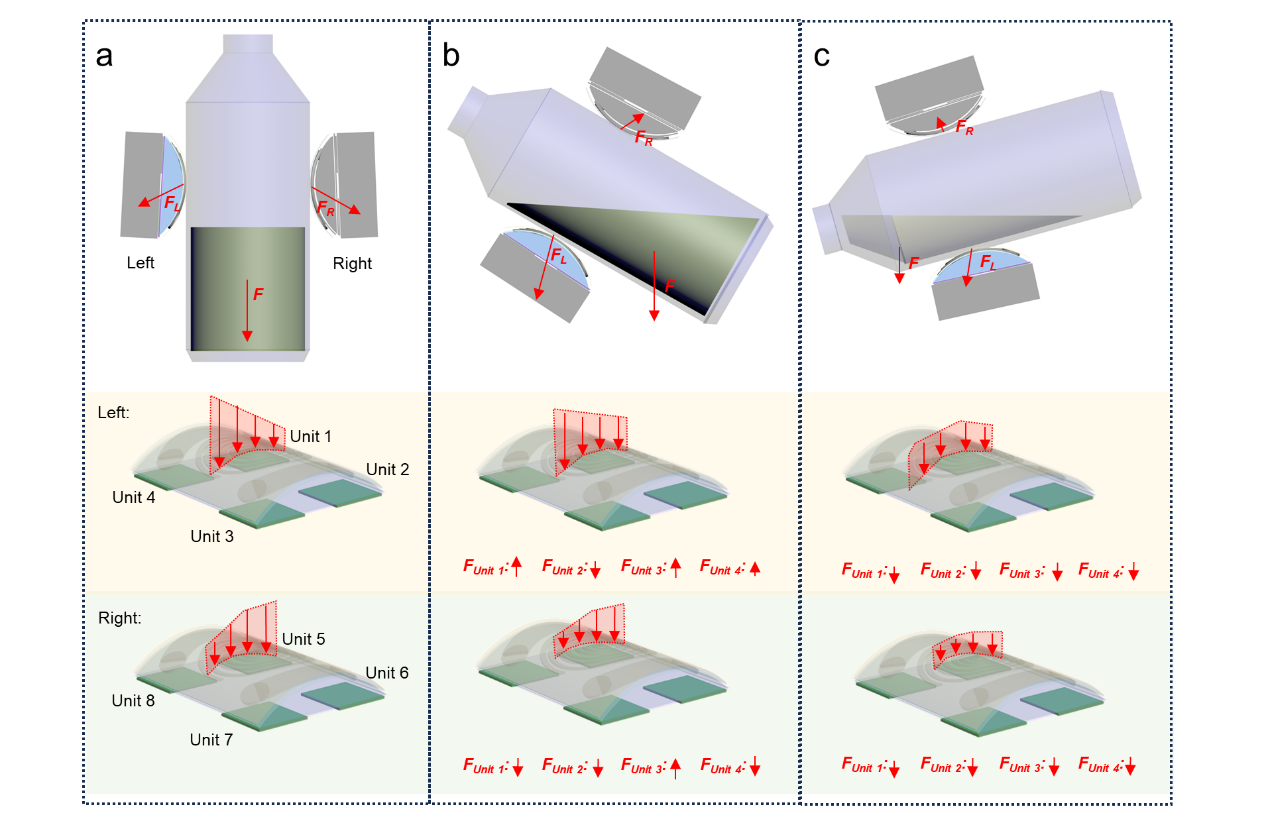
Figure S35 Force variations of the sensor array as the manipulator performs the pouring action.

**Table S1.** Numbers and names of 26 textiles

| Number | Name | Number | Name | Number | Name |
| --- | --- | --- | --- | --- | --- |
| A | Jacquard Suit Fabric | J | Extra Dense American Mesh | S | Cotton White Fabric |
| B | DuPont Fabric | K | Knitted Ribbed Fabric | T | Tencel Twill |
| C | Laser Organza Fabric | L | Monofilament Cotton Toothpick Strip Fabric | U | Gold velvet fabric |
| D | Mercerized Fabric | M | Extra Thick Slant Washed Cotton | V | Chiffon |
| E | Issey Miyake Pleated Fabric | N | Enzyme washed ragged jacquard denim | W | Double-sided single-layer tweed |
| F | Hot Stamping Drawstring Fabric | O | Double Layer Double Side Smooth Wool Cashmere Fabric | X | Stretch bamboo cotton linen |
| G | Rocker Velvet Fabric | P | TPU rainwear frosted cloth | Y | Air Layer Space Cotton |
| H | Corduroy | Q | Silk Fabric | Z | Modal Fabric |
| I | Luminous Fabric | R | Simulated Silk Organza Fabric |  |  |
